# Measuring financial protection against catastrophic health expenditures: methodological challenges for global monitoring

**DOI:** 10.1186/s12939-018-0749-5

**Published:** 2018-05-31

**Authors:** Justine Hsu, Gabriela Flores, David Evans, Anne Mills, Kara Hanson

**Affiliations:** 1 Department of Health Systems Governance and Financing, World Health Organization, 20 Avenue Appia, 1211 Geneva, Switzerland; 2 World Bank, 3 Chemin Louis-Dunant, 1202 Geneva, Switzerland; 3 London School of Hygiene and Tropical Medicine, Keppel Street, London, WC1E 7HT United Kingdom; 4 London School of Hygiene and Tropical Medicine, 15-17 Tavistock Place, London, WC1H 9SH United Kingdom

**Keywords:** Catastrophic health expenditures, Financial protection, Health financing, Universal health coverage

## Abstract

**Background:**

Monitoring financial protection against catastrophic health expenditures is important to understand how health financing arrangements in a country protect its population against high costs associated with accessing health services. While catastrophic health expenditures are generally defined to be when household expenditures for health exceed a given threshold of household resources, there is no gold standard with several methods applied to define the threshold and household resources. These different approaches to constructing the indicator might give different pictures of a country’s progress towards financial protection. In order for monitoring to effectively provide policy insight, it is critical to understand the sensitivity of measurement to these choices.

**Methods:**

This paper examines the impact of varying two methodological choices by analysing household expenditure data from a sample of 47 countries. We assess sensitivity of cross-country comparisons to a range of thresholds by testing for restricted dominance. We further assess sensitivity of comparisons to different methods for defining household resources (i.e. total expenditure, non-food expenditure and non-subsistence expenditure) by conducting correlation tests of country rankings.

**Results:**

We found country rankings are robust to the choice of threshold in a tenth to a quarter of comparisons within the 5–85% threshold range and this increases to half of comparisons if the threshold is restricted to 5–40%, following those commonly used in the literature. Furthermore, correlations of country rankings using different methods to define household resources were moderate to high; thus, this choice makes less difference from a measurement perspective than from an ethical perspective as different definitions of available household resources reflect varying concerns for equity.

**Conclusions:**

Interpreting comparisons from global monitoring based on a single threshold should be done with caution as these may not provide reliable insight into relative country progress. We therefore recommend financial protection against catastrophic health expenditures be measured across a range of thresholds using a catastrophic incidence curve as shown in this paper. We further recommend evaluating financial protection in relation to a country’s health financing system arrangements in order to better understand the extent of protection and better inform future policy changes.

**Electronic supplementary material:**

The online version of this article (10.1186/s12939-018-0749-5) contains supplementary material, which is available to authorized users.

## Background

There is increasing interest in monitoring the impact of household health expenditures on living standards. This interest is growing because financial protection is a key component of universal health coverage (defined as everyone receiving the health services they need and protected from financial hardship in doing so), an agreed target for health in the Sustainable Development Goals (SDGs) [1]. Global-level monitoring is of particular interest as it allows benchmarking a country’s progress relative to others and encourages global dialogue and the exchange of country experience. Country-level monitoring is also of particular interest to understand progress over time or differences across sub-national levels, thereby helping to inform future policy reforms. The methodological analysis presented here is concerned with monitoring at the global level and focuses on comparisons across countries. Regardless of the level of monitoring, there is need for an indicator that leads to unambiguous assessments of comparative progress.

Monitoring financial protection typically relies on two indicators – catastrophic health expenditures associated with out-of-pocket (OOP) payments for health reducing people’s ability to spend on other essential items, and impoverishing health expenditures associated with OOP payments for health pushing or further pushing people into poverty. Both indicators are thus concerned with the impact of OOP payments, defined as those payments that patients make directly to health providers at the time of service. They include cost-sharing and informal payments (in kind and in cash) but exclude payments by a third-party payer [2]. This paper focuses on the former indicator of catastrophic health expenditures which monitors when OOP payments as a share of household resources reaches and/or surpasses a certain threshold. Choices in measuring this relate both to the definition of household available resources (denominator) and to the threshold (percentage) used to determine when the OOP share on health is catastrophic. There is no established gold standard for either, with considerable debate over the last decade. Earlier discussions focused on the definition of available household resources [3–5]. More recent discussions concerned the choice of the threshold [6–8]. In the absence of consensus, studies of catastrophic health expenditures frequently present results using multiple definitions of household resources and various thresholds [9–11].

For global monitoring to be meaningful for policy, it is important to understand if a country’s performance relative to that of another is insensitive to varying methodological choices. Does the assessment that a country has higher levels of financial protection than another depend on the method used to define available household resources? Does it also depend on the specific threshold? If the answer to one and especially to both is yes, then making sense of cross-country comparisons to draw conclusions about the relative performance of health financing systems becomes more challenging.

The objective of this paper is to assess the sensitivity of comparisons of country-level estimates of financial protection against catastrophic health expenditures to different methodological choices in indicator construction. In this analysis, sensitivity is assessed by the extent to which orderings of distributions of financial protection against catastrophic health expenditures across countries are consistent, irrespective of the threshold, and correlated, irrespective of the method for defining household resources. We adapted methods to test for restricted dominance. These methods have previously been applied in the measurement of poverty to assess sensitivity of poverty incidence rates to the choice of the poverty line [12], and have more recently been studied as a means to assess the sensitivity of the incidence of catastrophic health expenditures to the choice of the threshold [8, 13]. This empirical paper is one of the first to apply such methods to assess the impact of varying methodological choices on global monitoring of financial protection. It demonstrates whether these choices matter, provides new insight into challenges for monitoring, and recommends a way forward for measuring financial protection beyond conventional approaches.

### Conceptual underpinnings

The concept of financial protection rests on the theoretical foundations of insurance and the economic value of reduced uncertainty or financial risk of being exposed to large healthcare costs [14, 15]. Health insurance, whether run by governments, nongovernmental organizations, communities or commercial companies, seeks to reduce this risk for the individual; when a country’s health financing arrangements fail to adequately provide this insurance function, access to health services will either be foregone or privately financed through OOP payments. The concern of catastrophic health expenditures is with the negative impact that OOP payments can have on economic well-being, for example when an individual forgoes consumption of other necessities (e.g. food) to pay for health. It is identified by comparing OOP payments for health to some definition of household resources and whether these surpass a certain threshold.

Thus, in measuring catastrophic health expenditures, there are two methodological choices. The first is the definition of household resources available to pay for health services. The second is the threshold used to identify health expenditures as catastrophic.

Defining household resources follows two main approaches, differing in whether they account for non-discretionary spending [16]. In the ‘budget share approach’ household resources are defined in relation to a household’s total budget without distinguishing spending on necessities. This approach is easy to understand and requires no further calculation. A further advantage is that it is not dependent on household allocation decisions across discretionary and non-discretionary items. However, it fails to distinguish between populations who just manage to meet subsistence needs with little or nothing left for discretionary expenditures and richer groups who have more latitude in discretionary spending.

The ‘capacity-to-pay (CTP) approach’ addresses this limitation, recognising that poorer households spend a higher proportion of available resources on essential items than richer households. It thus defines household resources as net of such spending. The idea is that spending on other basic necessities should not be considered part of resources available for health. CTP can be defined in various ways but commonly includes a component related to food spending. One well-established method defines this as total expenditures net of all food spending [16]. While its calculation is simple, a limitation of this method is that it does not recognise that some food spending is discretionary. Another popular method, proposed by Xu et al. (2003) [17], approximates the non-discretionary part of food spending as average food expenditure per equivalent adult across households in the 45th–55th percentile of the food budget share distribution. When actual food spending is below this amount, CTP is defined as total expenditure net of actual food spending. Any expenditure above this fixed subsistence expenditure amount is considered discretionary and available for spending on other goods and services, including health. These two CTP methods are conceptually similar but the latter adopts a stricter assumption of what is non-discretionary. It could thus be argued to more accurately estimate CTP of populations at the bottom of the income distribution. Critics of the Xu et al. (2003) [17] method argue that its definition of subsistence expenditure is not based on a normative standard (e.g. a food consumption basket) and that it can mean that a poorer household is judged to have greater CTP than a richer one^1^ [18, 19]. Further discussions on CTP approaches, including their conceptual underpinnings, exist elsewhere [20].

Other choices can be made in the definition of household resources. For example, whether this should be measured by consumption expenditure or by income [21], whether OOP should be included in the measure or netted out as it does not increase welfare [22], and whether other categories of expenditure, such as housing and utilities, should also be considered as essential in a CTP approach [23]. Measuring household resources using income was not explored in this analysis as the implications have already been studied elsewhere, and some seminal literature suggests that consumption is the preferable measure given it smooths fluctuations during periods of high and low income [24, 25]. Furthermore, it has been shown that the choice matters less when measuring national incidence rates of catastrophic health expenditures (as done in this paper) than when measuring inequalities across socio-economic groups [20]. We also do not consider the other two additional CTP variants in this paper as currently they are not as commonly used in the measurement of catastrophic health expenditures.

The second methodological choice in measuring protection against catastrophic health expenditure is the threshold used to define catastrophic OOP payments. Any such threshold is a normative choice. The choice is based on the idea that households who are spending above the threshold on health are left with a certain balance of their expenditure to spend on other essential items [15, 26]. Too low a threshold fails to capture a level of spending that causes households to forgo such items. Too high a threshold fails to capture small amounts of spending by the poor that are nonetheless catastrophic. Catastrophic thresholds in published studies typically vary between 10% and 40% depending on the definition of household resources, with a lower threshold used in the budget share method and a higher threshold in CTP methods [9, 27–31]. Typically a single threshold is uniformly applied across the population, but it can also vary such that a lower threshold is used for the poor and a higher threshold for the rich [6, 7].

## Methods

This analysis relied on household expenditure survey data from a sample of 47 countries over 2000–2012 (Additional file 1). This convenience sample was composed of nationally representative household survey datasets which the authors had access to and which had information on total consumption expenditure, including on OOP payments for health. The dataset represents a diverse spectrum of countries at different levels of economic development including low-, middle- and high-income countries, countries belonging to all five United Nations regional groups, and countries with diverse financing arrangements ranging from insurance schemes run by governments, nongovernmental organizations, or communities. Data provided information on household-level consumption expenditure which was aggregated into three expenditure variables (total, food, health). Total expenditure was estimated from monetary and in-kind payments on all goods and services plus the monetary value of consumption of homemade products. Food expenditure included items purchased and consumed from own production. Health expenditure consisted of OOP payments made by individuals to health providers at the time of service. All data were quality checked for missing values of the three aggregated expenditure variables and for illogical values (e.g. total expenditure<food expenditure). The frequency of such observations was minimal and these were dropped from the dataset.

For each household in each country dataset, three health expenditure ratios were constructed as the share of OOP payments for health in total expenditure, total expenditure net of all food expenditure, and total expenditure net of subsistence expenditure on food (Table 1).

**Table 1:** Measuring catastrophic health expenditures

*Headcount ratio:* Share of the population spending τ% or more of household resources on OOP payments for health			
$$ \frac{\sum_h{m}_h{w}_h1\left( OOP\_{share}_h\ge \tau \right)}{\sum_h{m}_h{w}_h} $$			
*h* denotes a household			
*m*_*h*_ denotes the number of members of household *h*			
*w*_*h*_ denotes the sampling weight of household *h*			
1() is an indicator function which is equal to 1 if the condition is satisfied and 0 otherwise			
*τ* denotes a catastrophic threshold			
Approach	Budget share	Capacity-to-pay	
Method	Total expenditure	Non-food expenditure	Non-subsistence expenditure
OOP share	$\frac{oop}{\mathit{\exp}}$	$\frac{oop}{\mathit{\exp}- food}$	$\frac{oop}{\mathit{\exp}- se}$
	*oop*=OOP health payments		
	*exp*=total expenditure	*food*=food expenditure	*se*=subsistence expenditure

To analyse the extent to which country comparisons were sensitive to the choice of the catastrophic threshold, we adapted a restricted dominance approach described by Flores et al. (see Additional file 2) [8, 13]. The dominance approach was originally developed in the measurement of inequalities comparing differences between two Lorenz (or concentration) curves to determine if the cumulative distribution of income (or other variable of interest) is always above the other, indicating the more preferred distribution on welfare grounds because the degree of inequalities is unambiguously less. Since then, dominance has been applied in the measurement of poverty to overcome limitations given that comparisons of poverty levels are sensitive to the choice of the poverty line [32–34]. By examining distributions of income across a specified range of poverty lines, restricted dominance thus allows for ranking distributions of poverty levels that are insensitive to the choice of the poverty line. Dominance is said to be restricted as it pertains to part of but not the full income distribution (i.e. given the focus is on the poor, particular interest is on the lower part of the distribution). Restricted dominance for poverty can be visualised by plotting on the vertical axis the incidence rate for poverty associated with multiple poverty lines over a specified range of the income distribution which are plotted on the horizontal axis. The resulting cumulative distribution function has been referred to as a ‘poverty incidence curve’ [21]. Comparative assessments of poverty distributions thus exhibit restricted dominance when, no matter the choice of the poverty line within the defined interval, one distribution of the incidence of poverty is always below another distribution [12, 32]. In other words, as assessed through statistical tests, the poverty incidence curves do not cross.

Analogous to this application of dominance to the measurement of poverty, distributions of catastrophic health expenditures can also be examined for restricted dominance. Indeed, measurement of catastrophe is similar to that of poverty as both rely on a defined benchmark (a poverty line in the case of poverty and a threshold in the case of catastrophe), and both are focused on a specific part of the distribution (the lower distribution of income in the case of poverty and the higher share of OOP payments for health in household resources in the case of catastrophe). The distributions of catastrophic OOP shares can also be visualised by plotting incidence rates of catastrophic health expenditures against a range of thresholds, resulting in a curve first referred to by Wagstaff as a ‘catastrophic spending curve’ [35]. Such a curve corresponds to a descending cumulative distribution function (CDF) and is denoted as 1 − *F*_OOP_share_ where *F*_OOP_share_(*τ*) ≡ *Prob*(OOP_share **≤** *τ*).

Whether comparisons of country-level estimates of catastrophic health expenditures result in consistent comparisons where one distribution exhibits restricted dominance over the other is assessed through statistical tests (Additional file 2). Testing for restricted dominance is thus valuable as it enables consistent conclusions to be drawn regarding differences in financial protection across countries. For restricted dominance testing to be applied to a measure, it must hold a minimum of four properties akin to the axioms used in the poverty framework to group poverty indices [32, 36]. The different measures of financial protection described in Table 1 are (i) focused, insensitive to changes above a threshold; (ii) population invariant, insensitive to differences in population sizes due to adding an exact replicate of a population; (iii) anonymous, insensitive to interchanges in budget share levels; and (iv) Pareto-improving, indicating an increase in financial protection as household resources increase [8].

Our approach for assessing sensitivity through restricted dominance consisted of using an intersection-union type of test under the null hypothesis of non-dominance between the distributions of the OOP shares on health of two countries. Specifically, *H*_0_: ${\widehat{F}}_{\mathrm{OOP}\_\mathrm{share}}^A-{\widehat{F}}_{\mathrm{OOP}\_\mathrm{share}}^B=0$. In other words, we tested differences between each country’s share of the population with catastrophic health expenditures conducted at each threshold along their CDFs. Following Chen and Duclos (2008) [32] and Kaur et al. (1994) [37], we employed tests based on the minimum t-statistic approach over *τ* ∈ [*τ*^*min*^; *τ*^*max*^] of the t-ratios of the differences between the catastrophic spending curve (see Additional file 2). We did not test over the full 0–100% threshold range but over two partial ranges of 5–85% and 5–40% with a one percentage point difference such that testing occurred for a total of 81 and 36 points, respectively. Testing was restricted above the lower 5% tail of the distribution because the concern with catastrophic health expenditures is for large OOP payments for health relative to household resources. In addition, testing along the upper tail of the distribution was also restricted: initially to 85% because of a concern for the power of the test and need for a sufficient number of observations for conducting t-tests, and subsequently to 40% because this is the highest threshold commonly used in the literature. It is expected that as the range of testing decreases, the likelihood of dominance increases.

The null hypothesis of non-dominance was rejected at the 10% level if the absolute value of all observed t-statistics was greater than 1.645, the critical value of the t-distribution. Rejection was at the 10% level to account for fewer observations at tails of the distribution. In these instances, the alternative hypothesis of dominance was not rejected, implying that one country’s headcount ratio of catastrophic health expenditures is always statistically significantly below the other within the range of thresholds tested. Furthermore, if the t-statistic was positive[negative], we inferred Country A[B] dominance. Failure to reject the null of non-dominance could be attributed to either: (i) insignificance, intervals of where the CDFs were not significantly different (absolute value of the t-statistic less than 1.645) or (ii) intersection, intervals where the CDFs crossed at least once (absolute value of t-statistic greater than 1.645 and signs of the t-statistic changed for any given pairwise comparison).

**Fig. 1:**
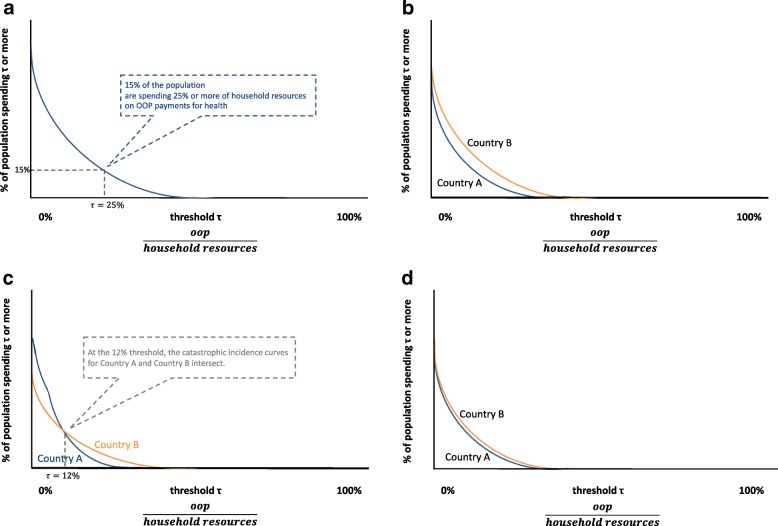
Illustration and interpretation of descending cumulative distribution functions for catastrophic health expenditures. All figures show a descending cumulative distribution function of OOP shares on health in household resources (also referred to as a ‘catastrophic incidence curve’). The y-axis represents the proportion of the population whose OOP shares on health in household resources meet or exceed threshold *τ*, and the x-axis shows the range of catastrophic thresholds *τ*. Any point on the curve can thus be interpreted as the incidence rate of catastrophic health expenditures for a given threshold. In (**a**), the cumulative distribution function shows that 15% of the population are spending 25% or more of household resources on OOP payments for health. In (**b**), Country A is said to exhibit dominance over Country B given its catastrophic incidence curve is always below that of Country A. In other words, the proportion of its population facing catastrophic health expenditures (y-axis) is always lower than Country B, no matter the threshold (x-axis). In (**c**), Country A and Country B exhibit non-dominance due to intersection given their catastrophic incidence curves intersect at the 12% threshold. This means that the proportion of the population in Country A facing catastrophic health expenditures is lower than Country B for thresholds below 12% but is higher than Country B for thresholds above 12%. In (**d**), Country A and Country B exhibit non-dominance due to insignificance given their catastrophic incidence curves differ but not to a statistically significant degree. This means that the proportion of the population in Country A facing catastrophic health expenditures differs from the proportion of the population in Country B facing catastrophic health expenditures but the difference is insignificant

Different dominance relationships are illustrated in Fig. 1. Figure 1a shows a descending CDF of catastrophic health expenditures for one country and describes how the CDF gives the probability of the population spending *τ* percent or more of household resources on health, where each point of a CDF is equivalent to the incidence rate of catastrophic health expenditures at threshold *τ* – referred to by Wagstaff as a ‘catastrophic spending curve’ [35]. Figure 1b illustrates a pairwise comparison resulting in dominance for the country exhibiting a lower CDF (i.e. lower levels of catastrophic health expenditures) and Fig. 1c and 1d resulting in non-dominance due to intersections and insignificance. Dominance and the type of non-dominance should ultimately be established through statistical tests.

For each method of constructing OOP shares on health, we assessed the frequency and proportion of comparisons exhibiting dominance (indicating cross-country assessments insensitive to the choice of the threshold) across all possible 2162 pairwise comparisons. A higher proportion of dominance is preferable as it increases confidence in the reliability of cross-country assessments. We also assessed the frequency and proportion of comparisons resulting in non-dominance (indicating assessments sensitive to the threshold) due to differences in CDFs found to be insignificant and non-dominance due to intersections of CDFs. Finally, we identified the longest continuous threshold range over which observed t-statistics were significant within each pairwise comparison and then averaged this across all comparisons for each method of defining household resources. A higher average length indicates a longer interval of dominance and suggests that the method is less sensitive to the threshold. The length can also be considered an indirect assessment of the overall power to test for dominance as a longer range of significance increases the ability to accept the alternative hypothesis of dominance.

The sensitivity of cross-country comparisons to methods for defining available household resources was also assessed. First, we compared the proportion of pairwise comparisons resulting in dominance when using each method. The higher the proportion of comparisons resulting in dominance, the less sensitive is that method for defining household resources compared to another. Second, we computed Spearman’s rank correlation coefficient of country rankings across each method. Rather than rank countries based on their incidence rate of catastrophic health expenditures at a single threshold, we ranked country distributions of OOP shares restricted to part of the catastrophic incidence curve over the popular 5–40% threshold range. Thus, for each method, countries were ranked by counting the number of pairwise comparisons for which the incidence rate of catastrophic health expenditures in one country was always statistically lower than another country over the entire popular threshold range of 5–40%, minus the number of pairwise comparisons for which the incidence rate was always statistically higher. If a pairwise comparison resulted in non-dominance, it was ignored since it does not allow for an unambiguous ordering of countries. The higher a country’s rank, the more frequently its incidence rates were lower than higher compared to other countries. This assessment thus indicates the sensitivity of country rankings to using different methods to define household resources.

## Results

Across all three methods for measuring catastrophic health expenditures, on average, just over a fifth (21.5%) of all possible 2162 country comparisons resulted in rejection of the null hypothesis in favour of the alternative hypothesis of dominance (Table 2). In other words, only 465 of the total 2162 country comparisons resulted in dominance or a consistent assessment of a country’s incidence rate of catastrophic health expenditures relative to another; in contrast, 1697 out of 2162 comparisons resulted in non-dominance or an inconsistent assessment such that, depending on the choice of the threshold, a country’s incidence rate was sometimes better and sometimes worse than another. Thus, a country’s assessment of financial protection relative to another was sensitive to the choice of the threshold over the 5–85% range. The degree of sensitivity to the threshold varied depending on the method for defining household resources. Following the budget share approach where OOP shares on health are constructed using total expenditure in the denominator, the null hypothesis of dominance was rejected for only 10.7% of comparisons (i.e. 232 of 2162 comparisons resulted in consistent assessments). This more than doubled when using CTP approaches but still remained low, increasing to 26.9% using non-subsistence expenditure and 27.0% using non-food expenditure (582 and 584 out of 2162 comparisons resulted in consistent assessments, respectively). In other words, at least three-quarters of comparisons were sensitive to the threshold, resulting in inconsistent assessments where either of the two countries was found to have higher and lower levels of financial protection depending on the threshold or where differences between two countries were not statistically significant.

**Table 2:** Analysis of dominance between country distribution functions of OOP shares on health

Approach	Budget share		Capacity-to-pay			
Method	Total expenditure		Non-food expenditure		Non-subsistence expenditure	
Threshold range	5–85%	5–40%	5–85%	5–40%	5–85%	5–40%
*Dominance relationship (frequency (proportion))*						
Dominance (restricted)	232 (10.7%)	1082 (50.0%)	584 (27.0%)	1202 (55.6%)	582 (26.9%)	1200 (55.5%)
Non-dominance due to insignificance	1352 (62.5%)	658 (30.4%)	830 (38.4%)	466 (21.6%)	838 (38.8%)	478 (22.1%)
Non-dominance due to intersections	578 (26.7%)	422 (19.5%)	748 (34.6%)	494 (22.9%)	742 (34.3%)	484 (22.4%)
Average length of dominance/Power of test	48.9	27.9	60.1	30.0	59.8	30.0

Dominance (restricted): one catastrophic incidence curve is always statistically above[below] another for a specified range of thresholds

Non-dominance due to insignificance: catastrophic incidence curves where the difference between curves is not statistically significant

Non-dominance due to intersections: catastrophic incidence curves that intersect and where difference between curves are statistically significant

Average length of dominance/Power of test: average continuous threshold range over which dominance was observed; considered an indirect assessment of the overall power to test for dominance

When assessing sensitivity by further restricting dominance testing to the popular 5–40% threshold range, the average proportion of robust assessments increased to approximately half of all comparisons. The budget share approach resulted in cross-country comparisons robust to the choice of the threshold 50.0% of the time, compared to the two CTP approaches which resulted in robust comparisons 55.6% and 55.7% of the time. Thus, when sensitivity was assessed by restricting dominance testing over the 5–40% threshold range, the choice of method for defining available household resources mattered less as sensitivities were of similar degrees.

As described in the methods section, cross-country comparisons resulting in non-dominance can be attributed to either catastrophic incidence curves that intersect or curves that differ from one another but not to a statistically significant degree. Intersections give inconsistent assessments of which country has statistically higher[lower] levels of financial protection depending on the threshold. Insignificances, while less informative in that they are unable to find statistically significant differences between two countries, do not result in contradictions. Dominance testing over the 5–85% threshold range indicated that cross-country comparisons following the budget share approach resulted in a slightly lower proportion of inconsistent comparisons or non-dominance due to intersections (26.7%) than either of the two CTP approaches (34.6% and 34.3%) (Table 2). When testing was further restricted to the 5–40% threshold range, the proportion of cross-country comparisons resulting in non-dominance due to intersections was slightly lower and similar whether using the budget share approach (19.5%) or either CTP approaches (22.9% and 22.4%).

Table 2 also shows the average length of a continuous range of threshold points over which significant t-statistics were found according to each method for defining household resources. The length of this interval indicates over what threshold range comparisons result in consistent assessments and is indicative of the degree of sensitivity of relative country assessments to methods for defining household resources in the denominator, as well as the power of the dominance test to reject the null hypothesis. Results showed that CTP approaches appeared to be less sensitive and had greater power than the budget share approach over the 5–85% threshold range as the average threshold range over which dominance or consistent results were observed was always greater (60.1 and 59.8 threshold points for non-food and non-subsistence, respectively; compared with 48.9 threshold points for total expenditures). When dominance tests were further restricted to the 5–40% range, the CTP approaches still appeared to be less sensitive and to have greater power than the budget share method. However, the difference was diminished as interval lengths were more similar, ranging from 27.9 to 30.0 threshold points.

**Fig. 2:**
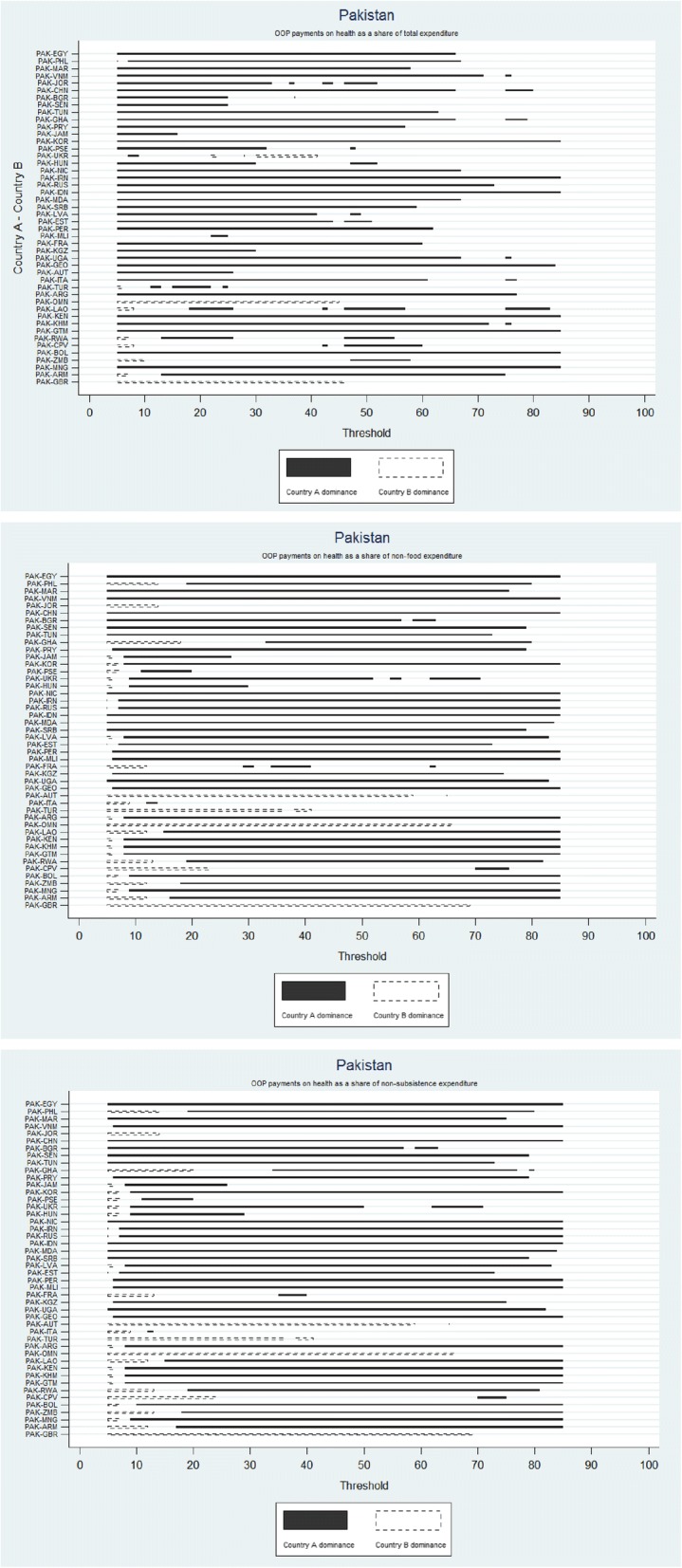
Example of sensitivity in cross-country comparisons to the choice of the threshold, observed through dominance. Each line represents a pairwise comparison of the incidence rates of catastrophic health expenditures between Country A and Country B. Countries are ordered by decreasing proportion of the population reporting any OOP. Solid bars indicate Country A dominance as it exhibited lower incidence rates of catastrophic health expenditures compared to Country B for the range of thresholds shown on the horizontal axis. Dashed bars indicate Country B dominance as it exhibited lower incidence rates of catastrophic health expenditures compared to Country A for the range of thresholds shown on the horizontal axis. White bars indicate that the difference between incidence rates of catastrophic health expenditures between Country A and Country B were not statistically significant for the range of thresholds shown on the horizontal axis. For any given pairwise comparison, one can therefore observe for which thresholds Country A has higher[lower] incidence rates of catastrophic health expenditures compared to Country B, and whether such assessments are sensitive to the choice of the threshold (i.e. if the type of bars displayed changes)

Additional file 3 shows results of dominance tests for each country for each of the three methods. Fig. 2 is shown here as an example, highlighting results for Pakistan. The x-axis shows the range of thresholds for when the share of OOP payments for health in household resources can be considered as catastrophic. The y-axis shows pairwise country comparisons between Pakistan and other countries. Solid bars reflect when Pakistan (Country A) exhibited a statistically lower incidence of catastrophic health expenditures) than another Country B. Dashed bars reflect when Country B exhibited lower incidence of catastrophe than Pakistan (Country A). The length of these bars reflects thresholds over which t-statistics used for assessing dominance were significant. Dominance was observed when a bar is continuously shown over the full threshold range of interest. Non-dominance due to intersecting CDFs was observed in lines with both types of solid and dashed bars, indicating that Pakistan exhibited both lower and higher incidence rates of catastrophe compared to Country B; and non-dominance due to insignificant CDFs was observed in lines with white space, indicating thresholds over which differences between Pakistan and Country B were insignificant. Figures thus show the degree of sensitivity to the choice of the threshold. For example, using the budget share method, comparisons of Pakistan were insensitive to the threshold with dominance shown by solid bars observed over seven countries with higher levels of financial protection no matter the threshold over the 5–85% range and over 28 countries over the 5–40% range. Some sensitivities to the threshold were observed in comparisons with Ukraine, Turkey, Laos, Rwanda, Cape Verde, Zambia, and Armenia with Pakistan observed to have higher incidence rates at lower thresholds (reflected by dashed bars) but lower incidence rates at higher thresholds (reflected by solid bars) for all comparisons except that with Ukraine where the opposite was observed. As seen here and in Additional file 3, the two CTP methods show almost identical profiles or degrees of sensitivities to the threshold.

When assessing sensitivity of cross-country comparisons to methods for defining household resources, all three methods were highly sensitive with at least three-quarters of comparisons dependent on the threshold (Table 2). Over the 5–40% range, comparisons were sensitive for approximately half of comparisons with similar degrees of sensitivity across methods. Sensitivity was also assessed by estimating Spearman’s rank correlation coefficients between country rankings of financial protection by each method (Table 3). The correlation was very strong between CTP methods using non-food and non-subsistence expenditure (*r* = .9963, *p* < .05), indicating nearly identical assessments of cross-country comparisons. In comparison, the correlation for each of these CTP methods with the budget share method was moderately strong (*r* = .7226 and *r* = .7171, *p* < .05).

**Table 3:** Correlation of country rankings of catastrophic health expenditure incidence rates over the 5–40% threshold range

	Total expenditure	Non-food expenditure	Non-subsistence expenditure
Total expenditure	1.0000		
Non-food expenditure	0.7226^*^	1.0000	
Non-subsistence expenditure	0.7171^*^	0.9963^*^	1.0000

Tested using Spearman’s rank correlation coefficient

^*^p < 0.05

## Discussion

This study is the one of the first published analyses to investigate the sensitivity of measuring financial protection against catastrophic health expenditures to varying methodological choices. These choices relate to the threshold used to identify health expenditures as catastrophic, causing a sacrifice of consumption on other essential items, and to the definition of living standards or household resources available to pay for health services. This paper is a methodological not a policy analysis and thus does not attempt to draw policy insight about the performance of any one country relative to another – the unit of analysis is methods of measurement rather than countries. In order for comparative assessments to be meaningful and to more effectively draw insight for policy, it is critical to understand how sensitive measurement is to these choices.

Defining the catastrophic threshold requires a choice. While more recent work has attempted to link this choice to disease outcomes or other factors of clinical relevance [38, 39], the choice of the threshold has also been referred to as arbitrarily defined [6, 40–42]. The choice would not be especially problematic if comparative assessments were insensitive to the threshold, but our results indicated this was not the case. Across all methods for measuring catastrophic health expenditures, country comparisons were robust to the choice of the threshold in only a tenth to a quarter of all comparisons within the 5–85% threshold range. If comparisons were restricted within the 5–40% threshold range, the proportion of assessments insensitive to the threshold increased to approximately half of all comparisons across all methods, with slightly more for CTP approaches. These results signal a challenge for global monitoring given that sensitivity reduces the ability to confidently draw reliable conclusions from cross-country comparisons as, depending on the threshold, a country could be assessed to perform relatively better and worse than another.

Regarding the choice for how to define household resources, dominance results revealed that the degree of sensitivity of cross-country comparisons using either the budget share or the two CTP approaches were all highly sensitive with almost three-quarters of comparisons sensitive over the 5–85% range. It is important to note that some of the sensitivity in CTP approaches may be partially due to indicator construction. This is linked to differences in food spending patterns where populations at lower levels of socio-economic status in countries at lower levels of economic development will spend proportionally more on food then those at higher levels. While this by-product of CTP approaches may lead to intersections in catastrophic incidence curves, it is also why such approaches are sometimes preferred as this property does take into account differences in socio-economic levels of development and attaches greater concern to equity. When assessing sensitivity over the 5–40% range, these were sensitive to a similar degree across all methods with approximately half of comparisons sensitive, although CTP approaches were slightly less so. Correlations across country rankings were also moderate across those produced by the budget share and both CTP methods and were very strong between the two CTP methods. Thus, the choice of the denominator and whether non-discretionary expenditures should be considered part of household resources available to pay for health has less practical implications for measurement than the choice of the threshold. Furthermore, all sensitivity results from CTP methods using non-food and non-subsistence expenditure were nearly identical. Given the two methods are conceptually related, as both exclude some or all food expenditure from the estimate of resources available to spend on health, the non-food method is preferable of the two as it is easier to compute and understand. The choice to define household resources following either the budget share or a CTP method represents an important normative choice. This is because CTP approaches are typically motivated by an ethical concern for the poor, given it recognises that poorer households spend a higher proportion of resources on essential items.

We recommend that the measurement of financial protection against catastrophic health expenditures should not be pinned to a single threshold as country assessments are sensitive to this methodological choice. In addition, financial protection has gradations of coverage rather than the simplistic protected or not protected categorisation offered by a single threshold. Measuring catastrophic health expenditures using only one point would result in a significant loss of information, failing to capture different degrees of hardship. The impact of OOP payments for health is not discrete but rather the financial burden they impose lies on a continuum from a very low burden where the impact is marginal, to a moderate burden where the impact may render access to some care unaffordable, to a very high burden where OOP payments cause severe financial hardship. Thus, measuring across multiple thresholds offers a more nuanced picture of the varying intensity of financial hardship due to paying for health by a population.

We further recommend that financial protection against catastrophic health expenditures be measured across a range of thresholds using catastrophic incidence curves as shown in this paper. Doing so would provide valuable policy insight as different health financing policies will impact different levels of OOP payments. For example, a country with specific policies for reducing copayments for inpatient services will provide more financial protection at higher thresholds of catastrophic health expenditures but not necessarily at lower thresholds. Specific policies tailored to country contexts might therefore explain why cross-country comparisons of financial protection are so sensitive to the threshold. It is important to evaluate how and why the system produces those patterns – analysing the pathways and interactions between policy interventions and health financing arrangements (e.g. prepayment and pooling, definition of the benefit package, cost-sharing arrangements, provider incentives) and how these influence financial protection. Financial protection is likely to improve if fragmentation in the financing system is reduced because risks are then increasingly pooled across the rich and poor and across the healthy and sick; if the definition of a benefit package is better defined to meet population health needs; if cost-sharing arrangements no longer allow for balanced billing; if referral systems are strengthened; and if perverse incentives inherent in open-ended fee-for-service provider payment methods are addressed. Indeed, the value of measuring financial protection is to not only assess the impact of OOP payments for health on living standards but also to understand how a country’s health financing system performs in terms of fulfilling an insurance function, linking back to the theoretical foundations of financial protection in health insurance. Indeed, Wagstaff et al. (2018) found that population coverage by a health insurance scheme is not strongly associated with the incidence rate of catastrophic health expenditures [43], further warranting investigation into design and implementation issues of insurance schemes for their influence on financial protection.

This study relied on a novel application of the dominance approach to studying distributions of OOP shares on health across a range of catastrophic thresholds. Other methodological studies have focused on either the choice of threshold or definition of living standards. Two studies by Ataguba et al. (2012) [6] and by Onoka et al. (2011) [7] examined implications when a single catastrophic threshold was applied uniformly and when it was varied to increase as a function of income, questioning the assumption that the threshold should be constant across rich and poor individuals alike. They found that allowing the threshold to vary increased estimated levels of catastrophic health expenditures [6, 7]. While these studies examined the application of the threshold, they did not address the percentage at which the threshold should be initially set. Other publications have focused on the definition of available household resources [4, 27, 30]. Wagstaff and van Doorslaer (2003) [27] developed and compared indicators following the budget share and CTP approaches, discussing their underlying theoretical concepts. Xu et al. (2003) [4, 30] further focused on CTP approaches and proposed a method motivated by a concern for fairness. These studies are noteworthy for developing methods popularly used today, but the evidence base for understanding the impact of varying the catastrophic threshold and the definition of household resources is still currently limited.

Key strengths and limitations of this analysis merit discussion. This study is one of the first analyses of the sensitivity of financial protection measures that relies on household expenditure surveys across many countries. Previous studies using expenditure surveys were done to produce global estimates or to assess determinants of financial protection but did not examine sensitivity across methods [30, 31, 43, 44]. Other multi-country studies with a methodological objective relied on health-specific surveys [8, 19]. Evidence shows that expenditure surveys provide more accurate estimation of OOP payments for health relative to other spending than if such data were collected from health-specific surveys [45].

A second strength of this study is that it comprehensively examined the impact of methodological choices in both the threshold and definition of household resources. First, sensitivity to the choice of the threshold was examined using a dominance approach. Second, sensitivity to the choice of the denominator in OOP shares on health was examined through comparisons of dominance results and correlation tests of country rankings of financial protection based on different methods for defining household resources. This study thus adds to the evidence base in a way not previously done. Given there is no unanimity on a gold standard nor understanding as to whether these choices ultimately matter, we provide new insight regarding the impact of methodological choices by empirically estimating the impact of varying methodological choices.

A first limitation of this analysis is that our analysis examined only a sample set of 47 surveys. It is important to keep in mind that the purpose of this analysis was not to conduct a comparative analysis of country performance but rather to assess sensitivity of country performance to methodological choices, such that country comparisons are not conclusive but illustrative. The time period of surveys between 2002 and 2012 may be considered as broad, however other global studies on financial protection presented analyses covering a similarly broad time period of 10-years [43, 44].

A second limitation is that the analysis relied on survey instruments that varied in design. While differences in survey design (e.g. recall period, number of expenditure items) will influence estimates of expenditures, the total effect is unclear [45]. Moreover, the same set of data from varied survey instruments was consistently used in all sensitivity analysis. It should also be noted that these surveys were all developed by national statistical offices and are systematically used to collect and categorise information on household expenditure. Such information is then consistently used for calculating consumer price indices and for measuring poverty rates. Thus, such variations in survey design are also a common issue in the global monitoring of other indicators, including poverty which is also derived from the very same income or expenditure surveys. To date, there is no established method for standardising survey design features in the collection of data on health spending.

This study explored methodological choices related to conventional indicators of financial protection popularly used in the literature and also adopted by international monitoring frameworks [1]. Such indicators do not capture the ex-ante value of reduced risk but rather the ex-post financial burden faced because of the lack of protection. Despite popular use in the empirical literature, these indicators have been criticised as being too narrow [41, 46] and are likely to underestimate the broader adverse effects of OOP payments for health given its focus on direct medical costs and exclusion of indirect costs such as those related to transport for accessing services, loss of income due to illness, or coping strategies.

## Conclusions

Global monitoring of financial protection against catastrophic health expenditures is challenging because there are different measurement methods and no established gold standard. To understand the challenge this might pose in global monitoring, we examined the sensitivity of cross-country comparisons to threshold points and to measures of living standards defined by household resources. We found moderate to high levels of sensitivity to the threshold such that drawing policy insight from cross-country comparisons should be done cautiously as interpretations could be limited if based on a single threshold point. The sensitivity of comparisons to the definition of household resources was also moderate to high but was similar across methods, although CTP approaches showed slightly less sensitivity. Hence, from a measurement perspective, our findings clearly demonstrate that the choice of the threshold matters most.

More valuable insight for policy can be gained by measuring across a range of thresholds using a catastrophic incidence curve as shown in this paper. This will allow for assessing whether the financial burden is marginal at lower thresholds of OOP shares on health, and/or more severe at higher thresholds. An area for future research is the application of such a measurement approach over time in a single country. In addition, such an evaluation should seek to evaluate not only changes in the catastrophic incidence curve but also how these are linked to specific policy intervention(s) and their interactions with the underlying health financing system. Identifying and assessing in which parts of the catastrophic incidence curve changes are occurring and how and why these might be linked to specific changes in health financing arrangements can ultimately help improve the design and implementation of related policies. Indeed, the point of measuring catastrophic health expenditures is not only to monitor the impact of OOP payments on living standards, but to also evaluate how a country’s health financing system can improve financial protection.

## Additional files

Additional file 1: List of surveys. (DOCX 17 kb)

Additional file 2: Technical note on the application of restricted dominance methods to catastrophic health expenditures. (DOCX 21 kb)

Additional file 3: Results of restricted dominance tests. (DOCX 3131 kb)

## Footnotes

1 This will occur for those households with similar levels of total expenditure where actual food spending by the poorer household is just below the fixed subsistence expenditure amount and that by the richer household is just above. This is because CTP is defined as total expenditure net of actual food spending when actual food spending is less than subsistence expenditure.
