# Supplementary material for: Measuring financial protection against catastrophic health expenditures: methodological challenges for global monitoring
Source: Int J Equity Health. 2018 May 31;17:69. doi: 10.1186/s12939-018-0749-5 (PMC5984475; doi:10.1186/s12939-018-0749-5)
Supplement: Supplementary file 1 — List of surveys. (DOCX 17 kb) [file 12939_2018_749_MOESM1_ESM.docx]

**Additional file 1: List of household surveys**

| **Country** | **Code** | **Survey** | **Year** |
| --- | --- | --- | --- |
| Argentina | ARG | Encuesta de Impacto Social de la Crisis Argentina | 2002 |
| Armenia | ARM | Integrated Living Conditions Survey | 2001 |
| Austria | AUT | Household Budget Survey | 2000 |
| Bolivia | BOL | Living Standards Measurement Survey | 2002 |
| Bulgaria | BGR | Integrated Household Survey | 2000 |
| Cabo Verde | CPV | Income and Expenditure Survey | 2001 |
| Cambodia | KHM | Cambodia Socio-Economic Survey | 2007 |
| China | CHN | Income and Expenditure Survey | 2000 |
| Egypt, Arab Rep. | EGY | Household Income Expenditure and Consumption Survey | 2012 |
| Estonia | EST | Household Budget Survey | 2007 |
| France | FRA | Household Budget Survey | 2006 |
| Georgia | GEO | Household Budget Survey | 2008 |
| Ghana | GHA | Living Standards Measurement Survey | 2006 |
| Guatemala | GTM | Living Standards Measurement Survey | 2000 |
| Hungary | HUN | Household Budget Survey | 2000 |
| Indonesia | IDN | National Socio-Economic Survey | 2001 |
| Iran, Islamic Rep. | IRN | Household Income and Expenditure Survey | 2007 |
| Italy | ITA | Household Expenditure Survey | 2001 |
| Jamaica | JAM | Jamaica Survey of Living Conditions | 2001 |
| Jordan | JOR | Household Income and Expenditure Survey | 2006 |
| Kenya | KEN | Kenyan Health Expenditure and Utilization Survey | 2003 |
| Korea | KOR | Household Income and Expenditure Survey | 2008 |
| Kyrgyz Republic | KGZ | Household Budget Survey | 2004 |
| Lao PDR | LAO | Expenditure and Consumption Survey | 2008 |
| Latvia | LVA | Household Budget Survey | 2006 |
| Mali | MLI | Enquete Malienne sur l'Evaluation de la Pauvrete | 2001 |
| Moldova | MDA | Household Budget Survey | 2007 |
| Mongolia | MNG | Household Socio-Economic Survey | 2009 |
| Morocco | MAR | Enquete nationale sur les revenus | 2007 |
| Nicaragua | NIC | Encuesta Nacional de Hogares sobre Medicion de Niveles de Vida | 2001 |
| Oman | OMN | Income and Expenditure Survey | 2000 |
| Pakistan | PAK | Household Integrated Economic Survey 2010-2011 | 2010 |
| Paraguay | PRY | Encuesta Integrada de Hogares | 2001 |
| Peru | PER | Encuesta Nacional sobre Medicion de Niveles de Vida (LSMS) | 2000 |
| Philippines | PHL | Family Income and Expenditure Survey | 2009 |
| Russian Federation | RUS | Russian Longitudinal Measurement Survey Round XI | 2002 |
| Rwanda | RWA | Enquête intégrale sur les conditions de vie des ménages | 2010 |
| Senegal | SEN | Enquetes de Suivi de la Pauvrete au Senegal | 2011 |
| Serbia | SRB | Living Standards Measurement Survey | 2000 |
| Tunisia | TUN | Enquete national sur la depense, la consommation et la niveau de vie des menage | 2010 |
| Turkey | TUR | Household Budget Survey | 2008 |
| Uganda | UGA | Uganda National Household Survey II | 2003 |
| Ukraine | UKR | Household Budget Survey | 2006 |
| United Kingdom | GBR | Family Expenditure Survey | 2000 |
| Vietnam | VNM | Household Living Standards Survey | 2010 |
| West Bank and Gaza | PSE | Palestinian Households Expenditure and Consumption Survey | 2004 |
| Zambia | ZMB | Living Conditions Monitoring Survey | 2006 |
