# Supplementary material for: Measuring financial protection against catastrophic health expenditures: methodological challenges for global monitoring
Source: Int J Equity Health. 2018 May 31;17:69. doi: 10.1186/s12939-018-0749-5 (PMC5984475; doi:10.1186/s12939-018-0749-5)
Supplement: Supplementary file 2 — Technical note on the application of restricted dominance methods to catastrophic health expenditures. (DOCX 21 kb) [file 12939_2018_749_MOESM2_ESM.docx]

**Additional file 2: Technical note on the application of restricted dominance methods to catastrophic health expenditures**

To make a robust comparison of the incidence rates of catastrophic health expenditures between two (or more) countries, we draw on methods for testing dominance. There are different orders of dominance, and we focus on what is referred to as first order dominance^[[1]](#footnote-1)^. First order dominance is observed when rankings of cumulative distribution functions (CDFs) of a variable of interest are robust (i.e. one distribution is always statistically above[below] another). We also restrict dominance testing across a specified (not unlimited) range of the distribution. As such this paper is specifically concerned with testing for first order restricted dominance.

In this application of dominance techniques to catastrophic health expenditures, let us first denote the cumulative distributions function of the share of out-of-pocket (OOP) payments for health in household resources as $F_{OOP share}\left( \tau\right)\equiv Prob\left( OOP share\leq\tau\right).$ Given catastrophic health expenditures is concerned with the share of OOP payments for health *greater* than some threshold, it then follows that the cumulative distribution function of *catastrophic* OOP shares is ${1-F}_{OOP share}\left( \tau\right)\equiv Prob(OOP share\geq\tau)$. The resulting distribution corresponds to a descending cumulative distribution function (CDF) and has been referred to by Wagstaff (2008) [36] as the ‘catastrophic spending curve’. Then, the necessary and sufficient condition for the incidence of catastrophic health expenditures to be lower in country A than in country B for thresholds $\tau\in\left[ \tau^{min};\tau^{max} \right]$ with $\tau^{min}$>0 and $\tau^{max}$<1 is that ${1-F}_{OOP share}^{A}\left( \tau\right)\leq{1-F}_{OOP share}^{B}\left( \tau\right)$ over$\left[ 0;\tau^{max} \right].$ In other words, country A is said to stochastically dominate country B if $F_{OOP share}^{A}\left( \tau\right)\geq F_{OOP share}^{B}\left( \tau\right)$ over$\left[ 0;\tau^{max} \right]$.

To establish first order restricted dominance, statistical testing is needed. We use an intersection-union type of test under the null hypothesis of non-dominance between the distributions of the OOP shares of two countries. Specifically, $H_{0}$: $\hat{F}_{OOP share}^{A}-\hat{F}_{OOP share}^{B}=0$. Following Chen and Duclos (2011) and Kaur et al (1994), we employed tests based on the minimum t-statistic approach. We used the minimum over $\tau\in\left[ \tau^{min};\tau^{max} \right]$ of the t-ratios of the differences between the catastrophic spending curve, i.e. $t_{min}={min}_{\tau\in\left[ \tau^{min};\tau^{max} \right]}\frac{\hat{\Delta}\left( \tau\right)}{\hat{\sigma}_{\hat{\Delta}\left( \tau\right)}}$ with$\hat{\Delta}\left( \tau\right)=\hat{F}_{OOP share}^{A}-\hat{F}_{OOP share}^{B}$ and $\hat{\sigma}_{\hat{\Delta}\left( \tau\right)}$ corresponding to the estimate of the standard error on the estimator of $\Delta\left( \tau\right)$.

1. Higher orders of dominance are described and discussed in [12, 32, 34]. [↑](#footnote-ref-1)
