# Supplementary material for: Measuring financial protection against catastrophic health expenditures: methodological challenges for global monitoring
Source: Int J Equity Health. 2018 May 31;17:69. doi: 10.1186/s12939-018-0749-5 (PMC5984475; doi:10.1186/s12939-018-0749-5)

**Additional file 3: Results of restricted dominance tests**

**Note:** Countries ordered by decreasing proportion of the population reporting any OOP.


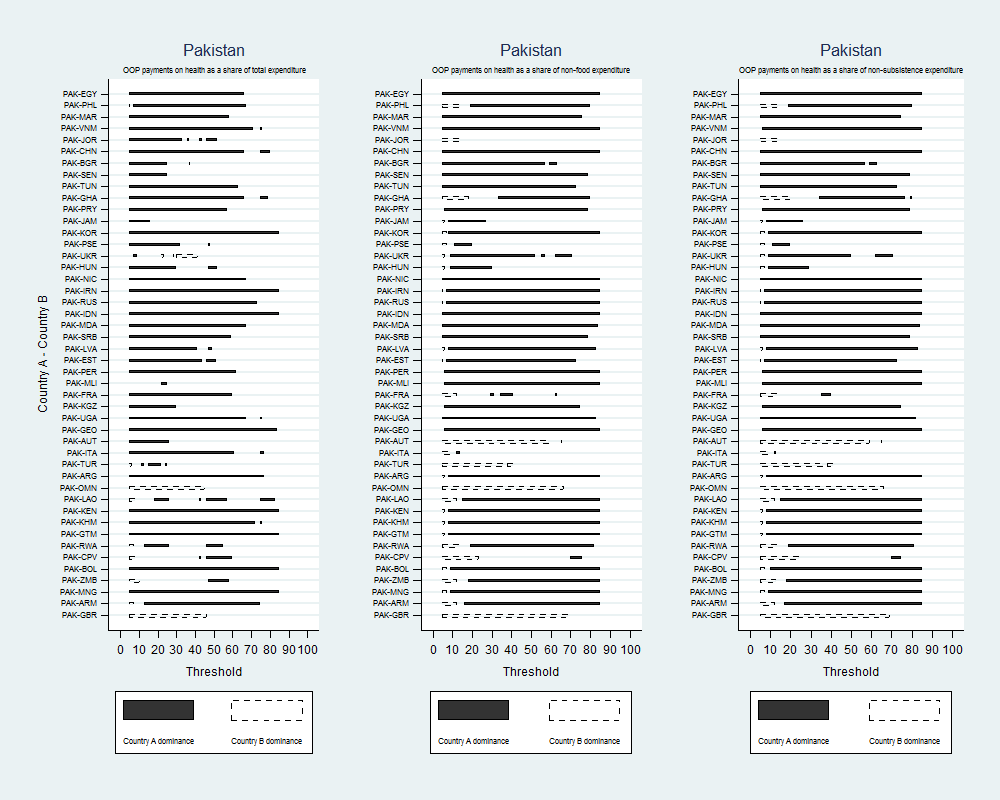


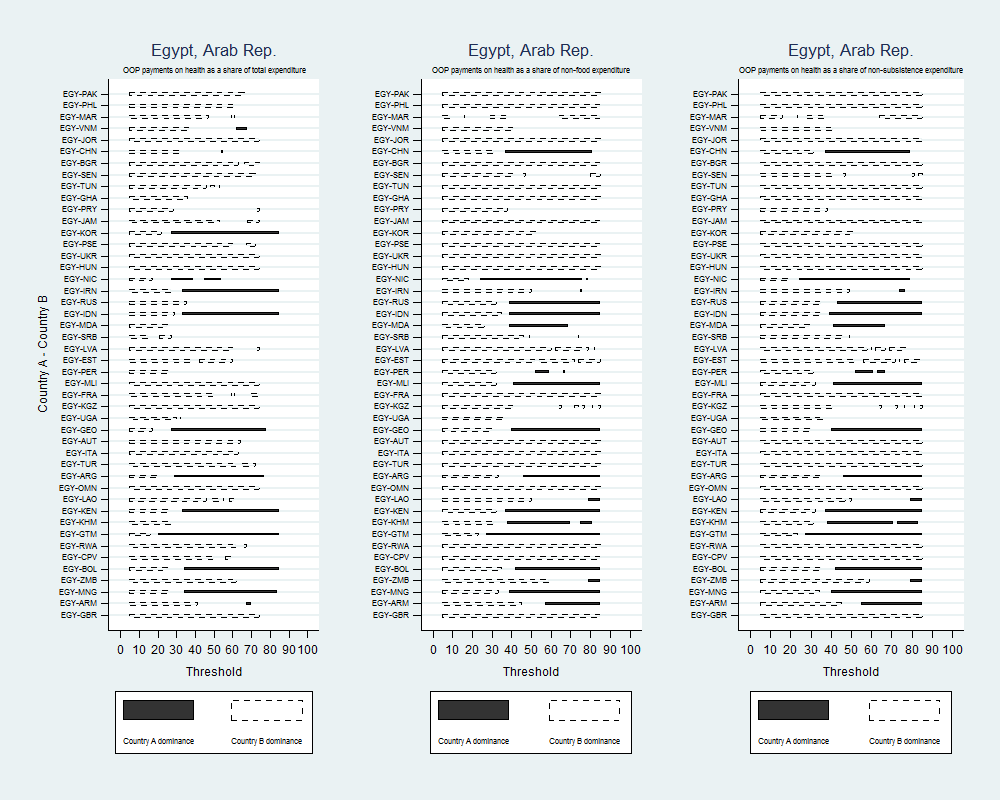


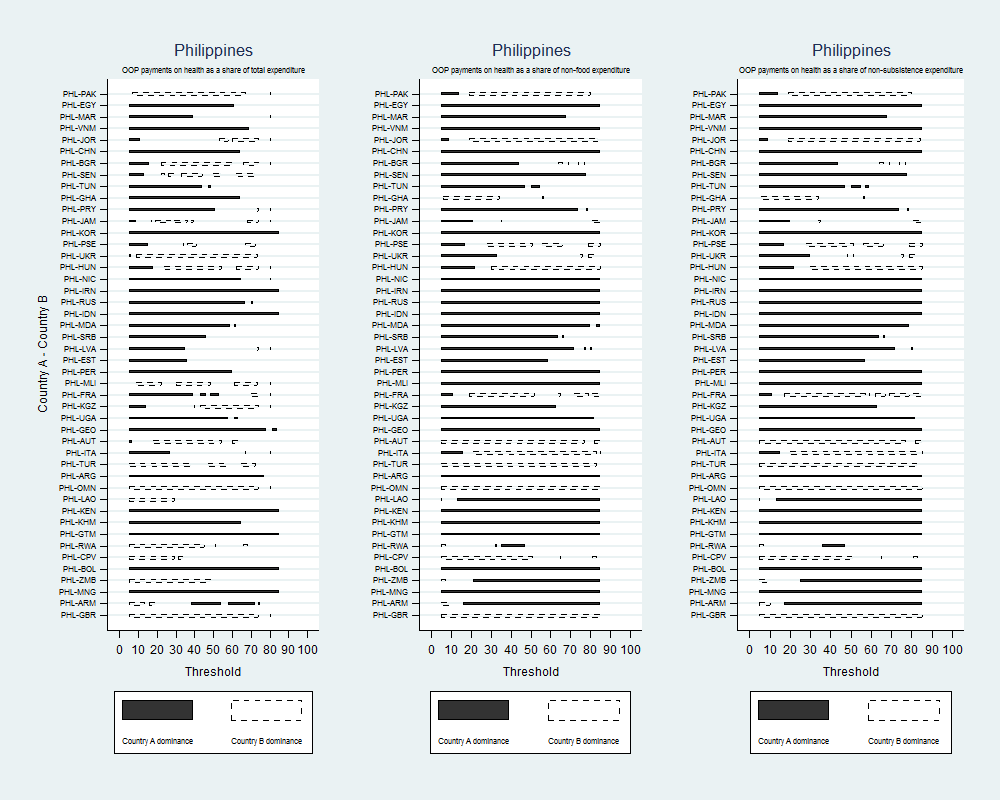


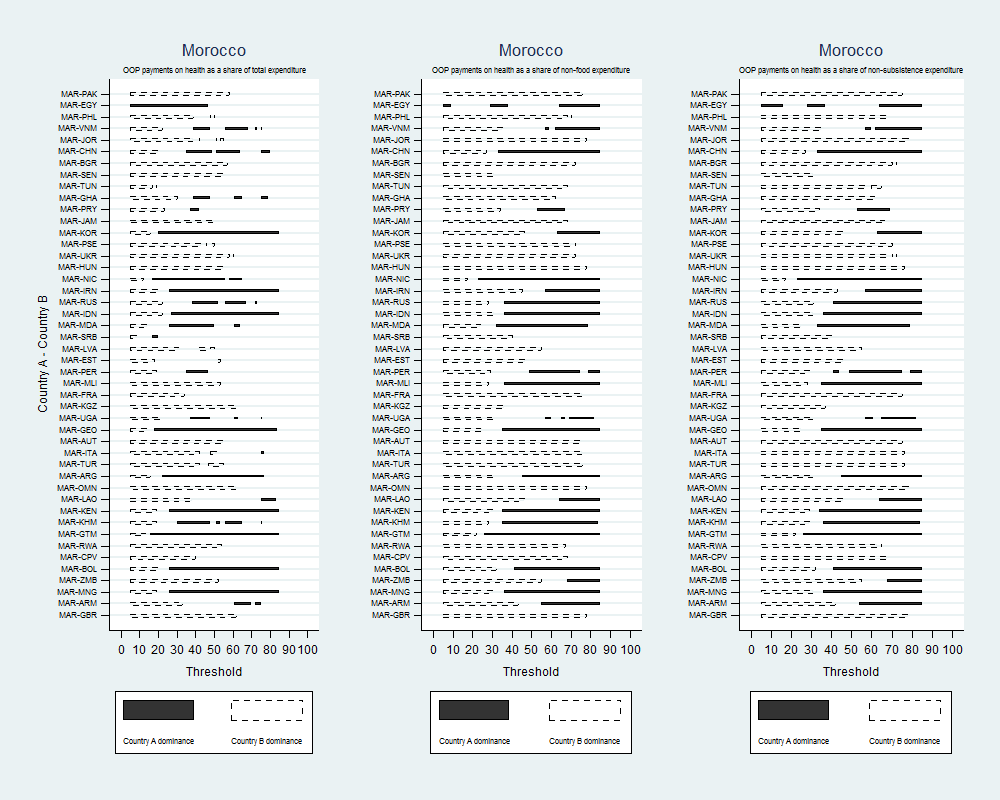


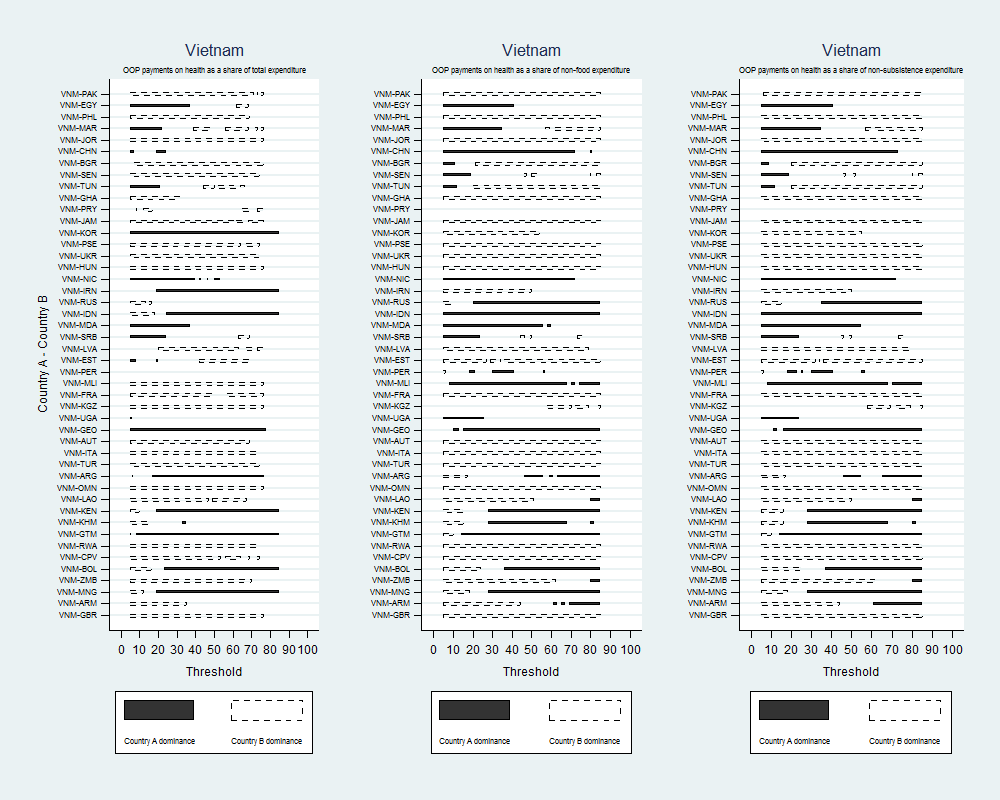


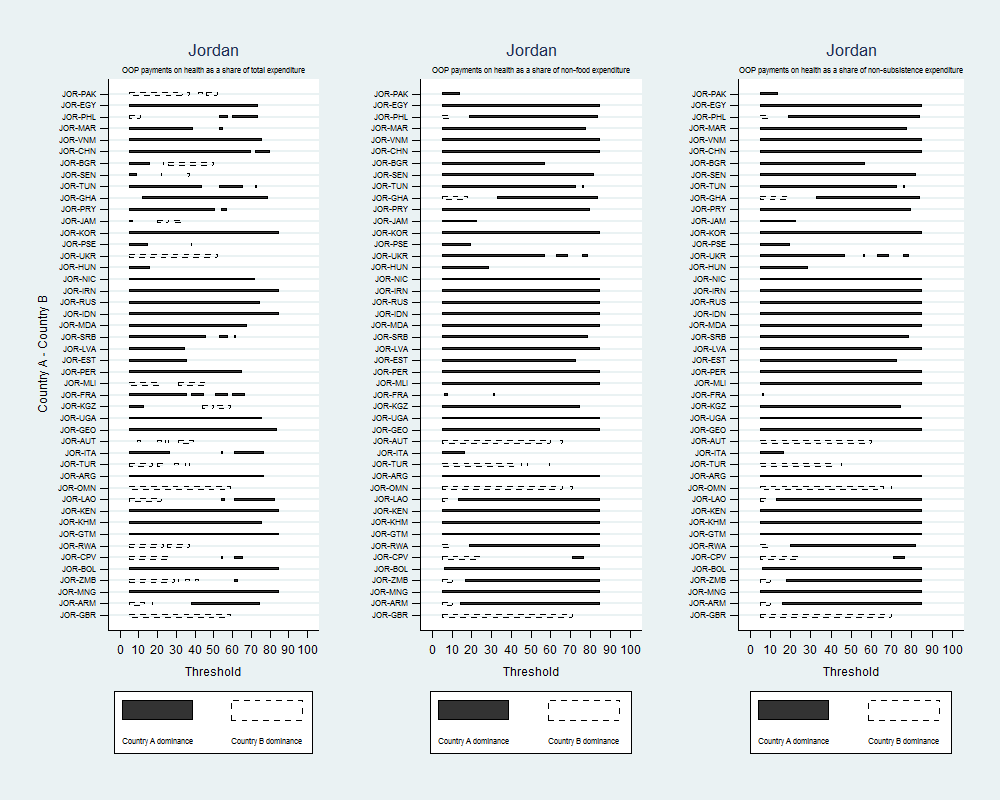


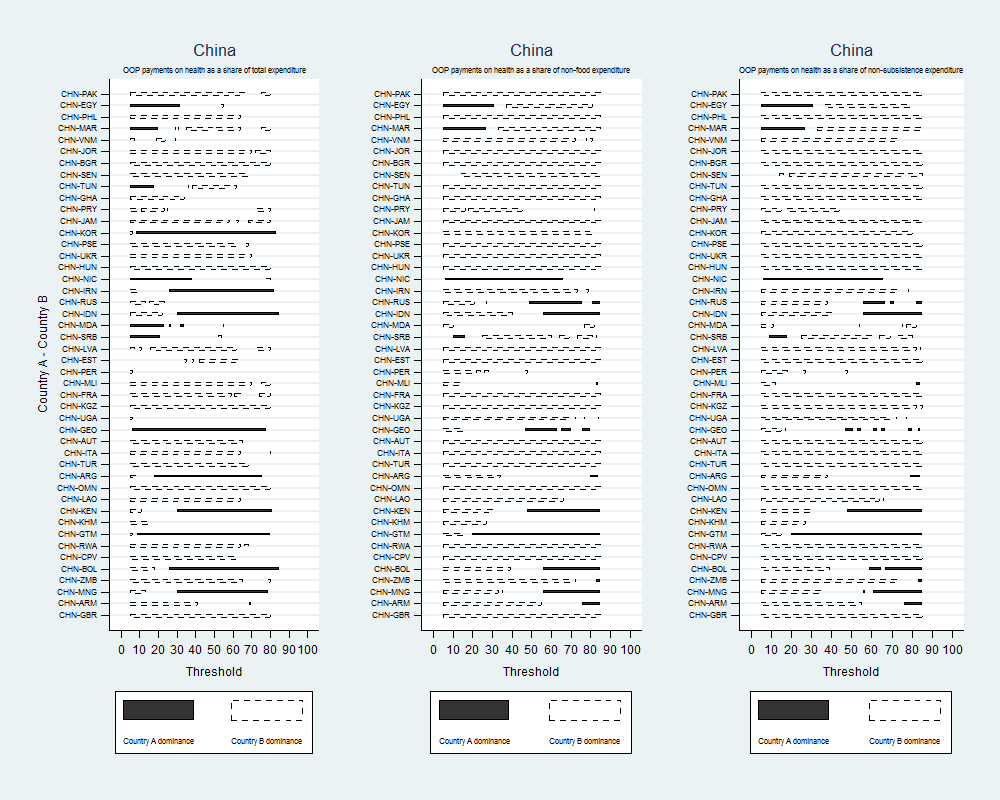


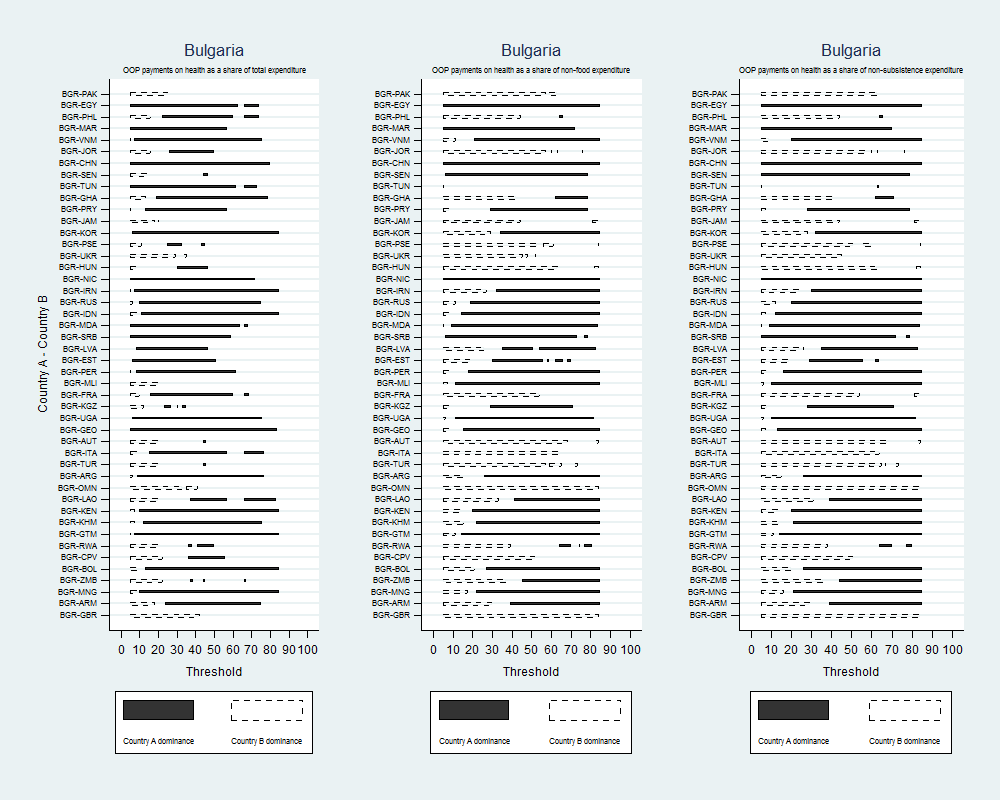


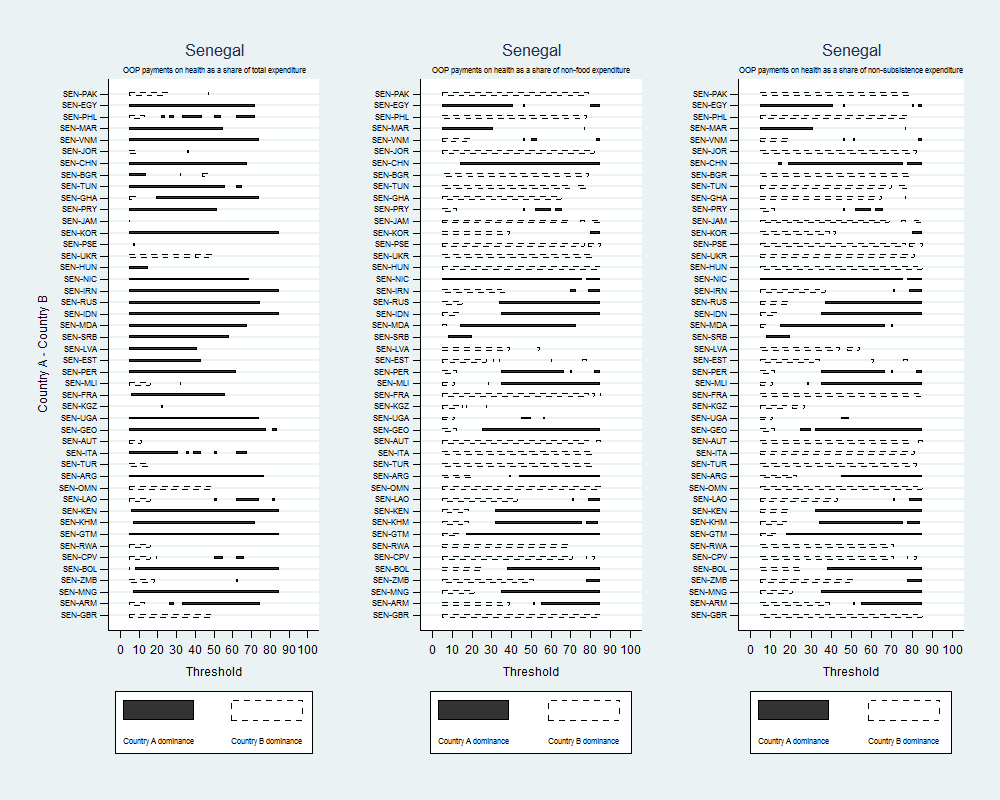


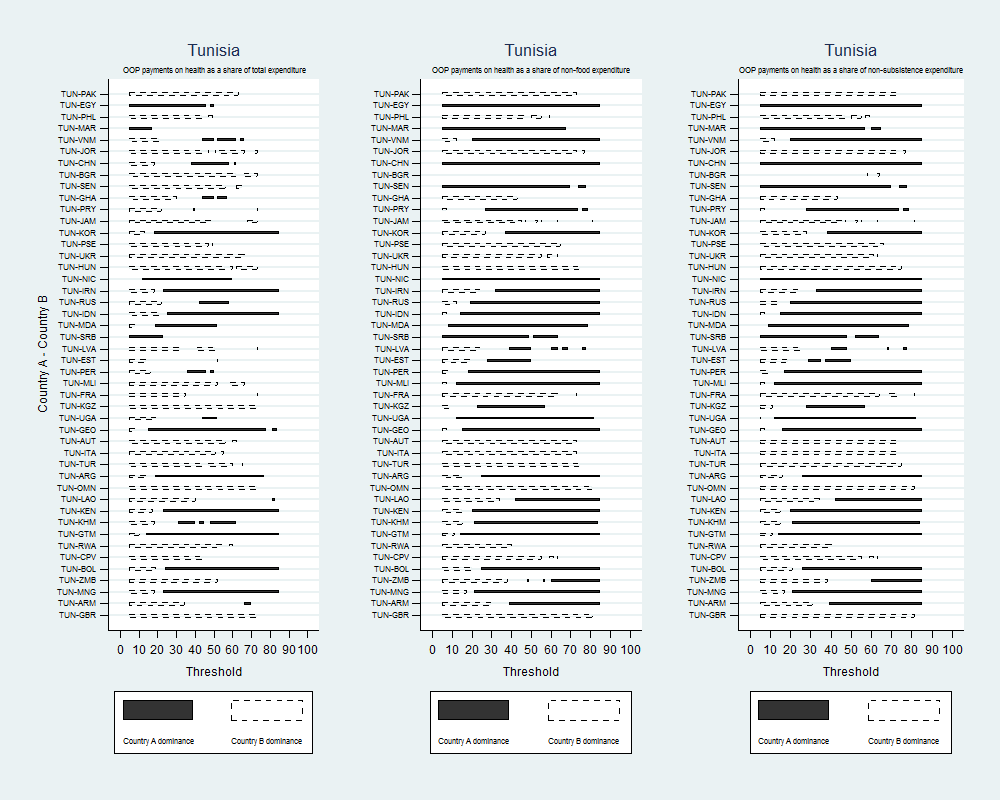


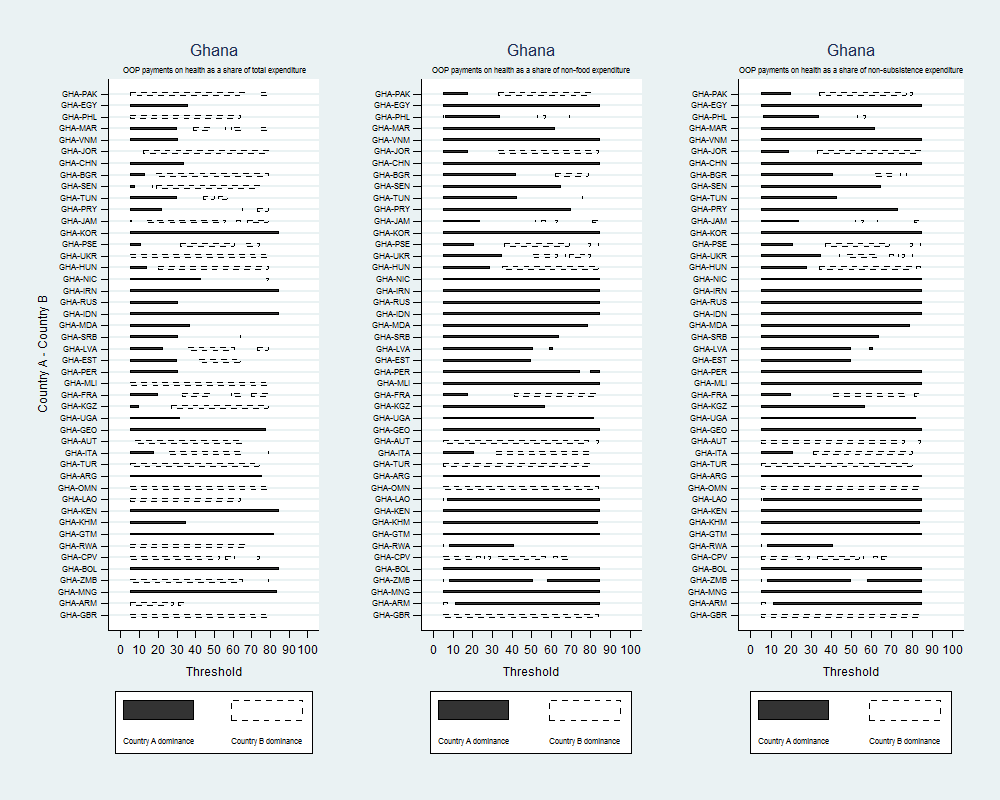


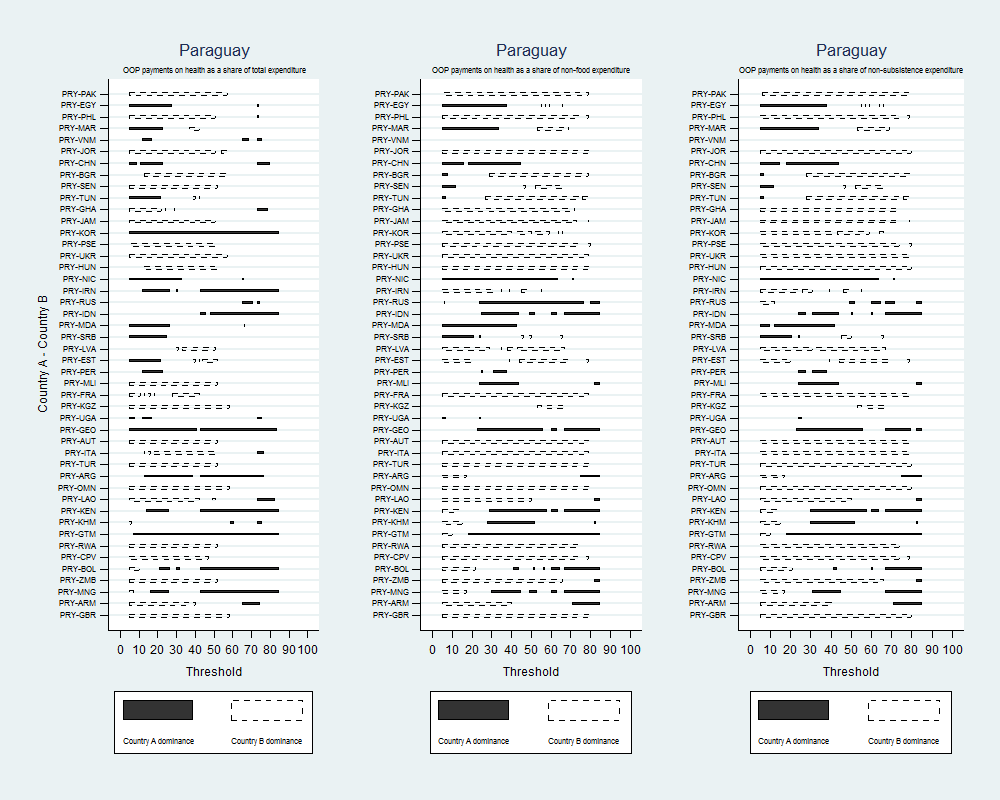


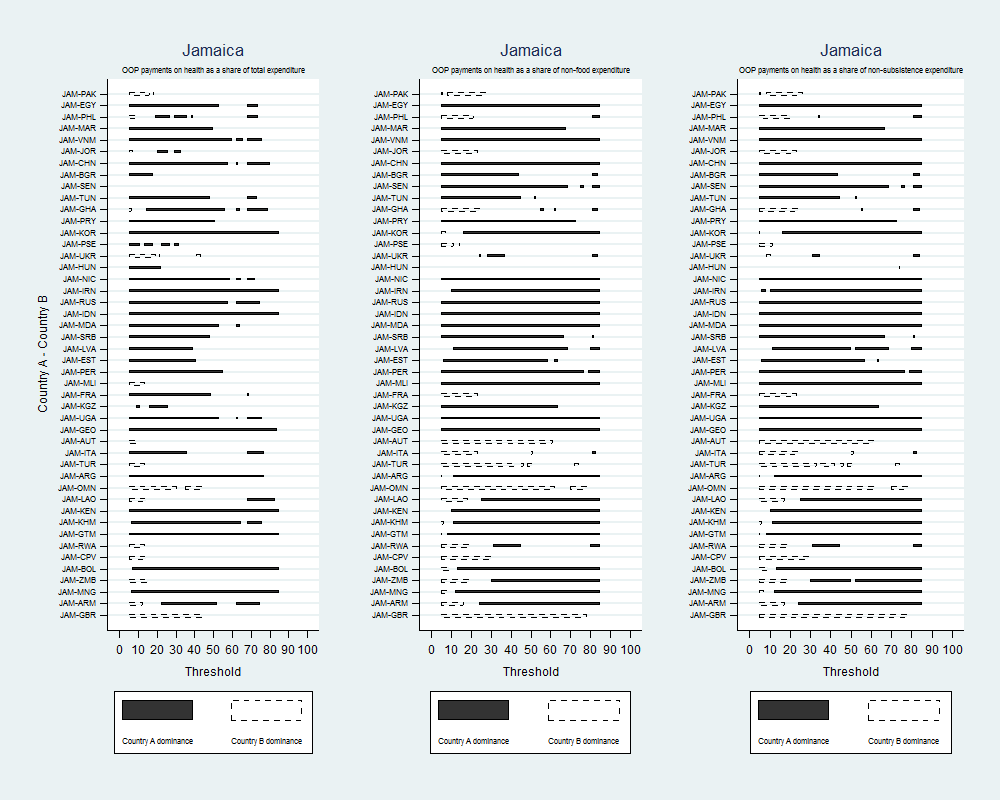


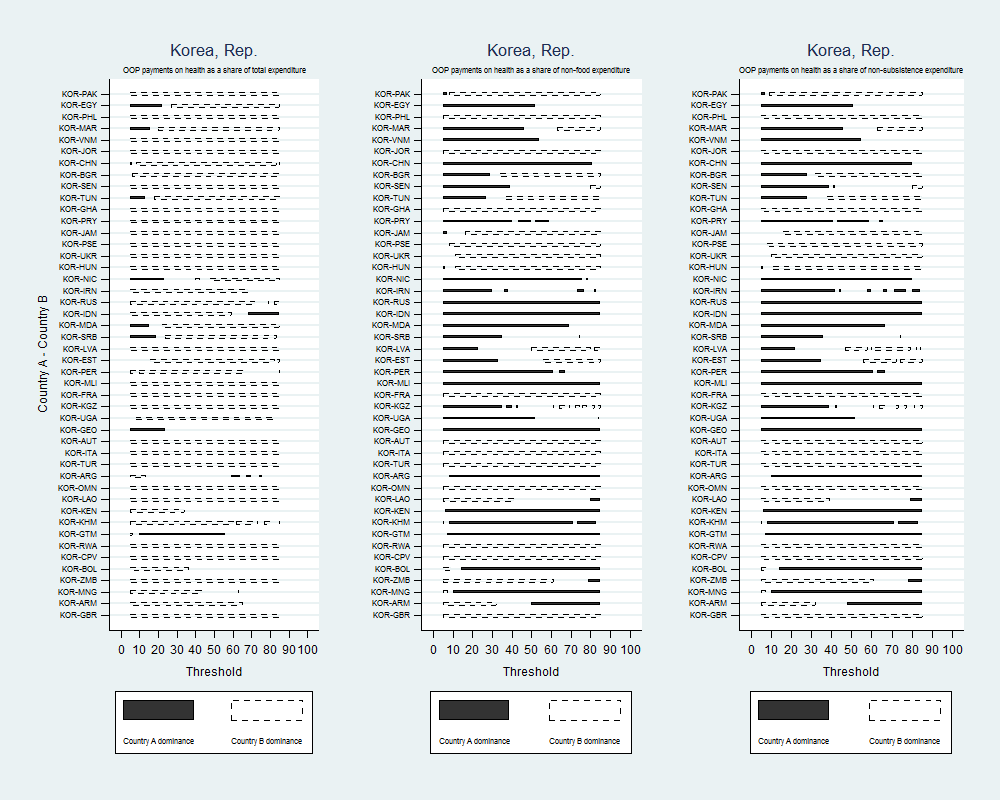


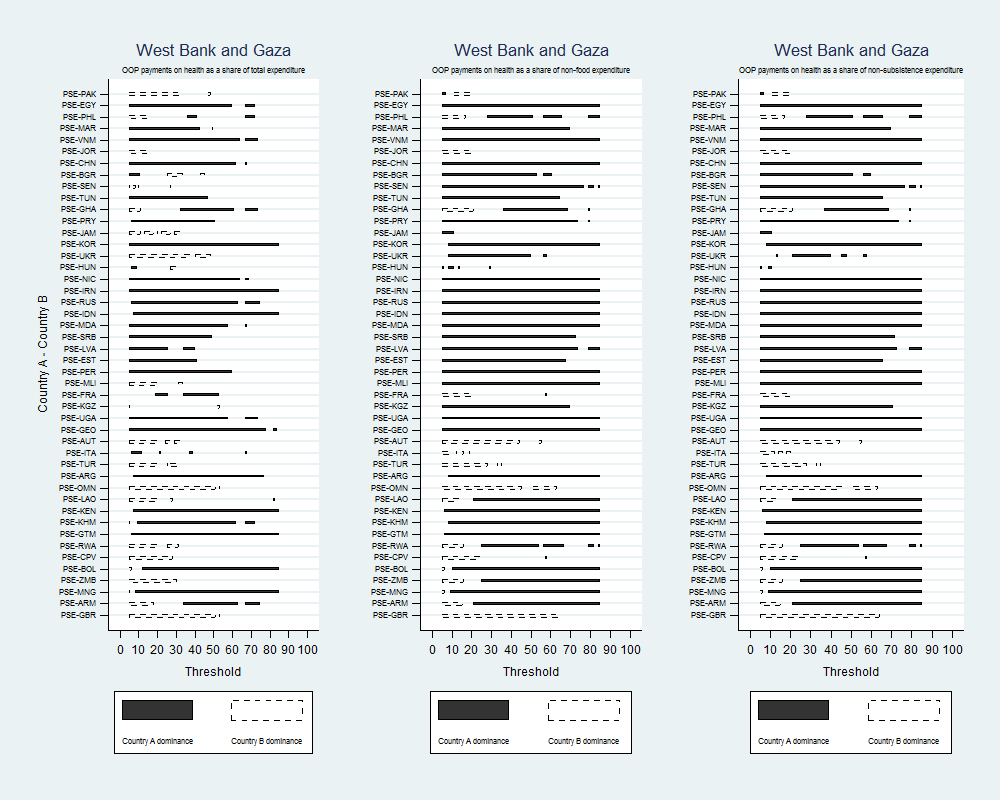


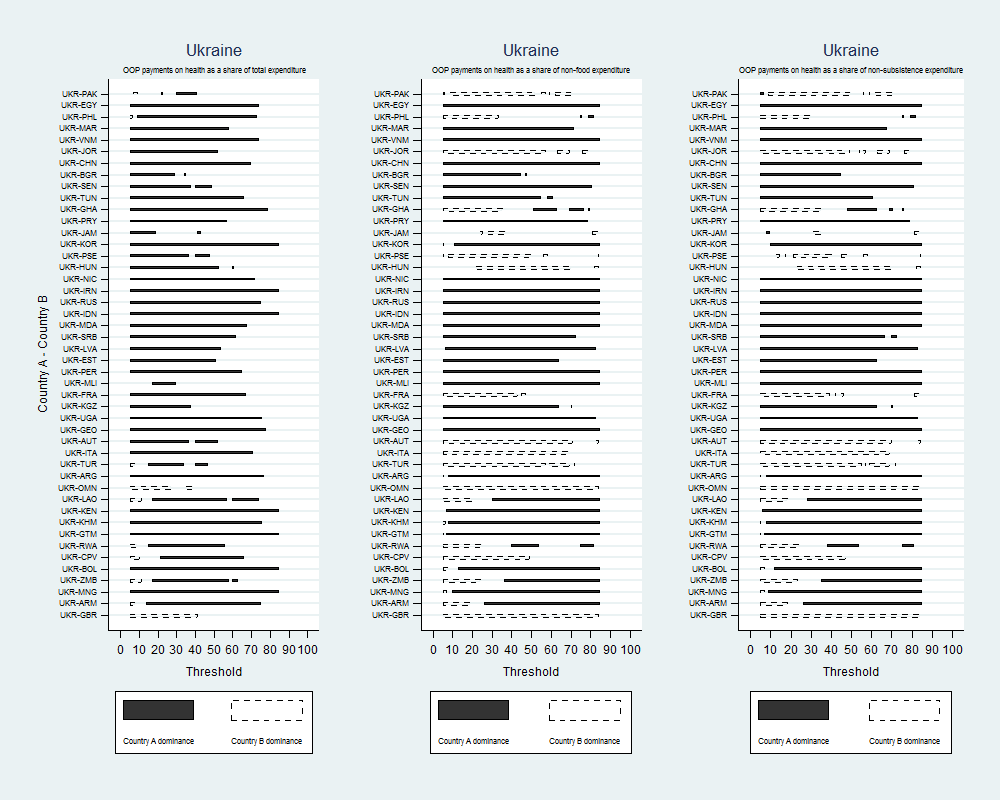


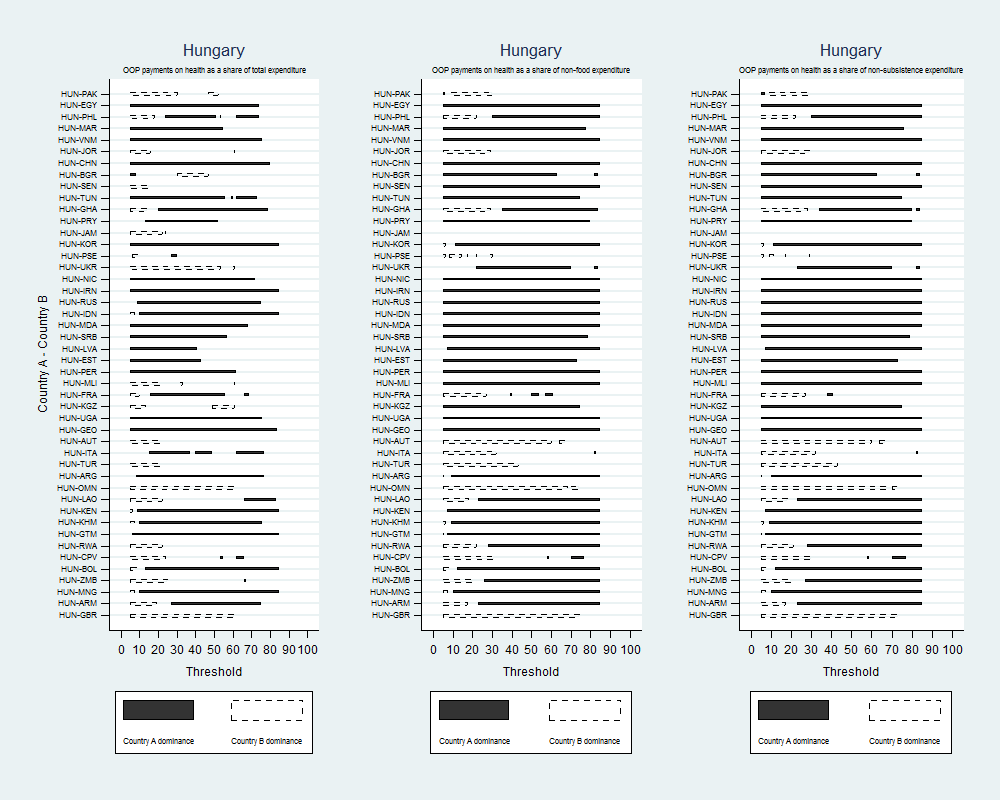


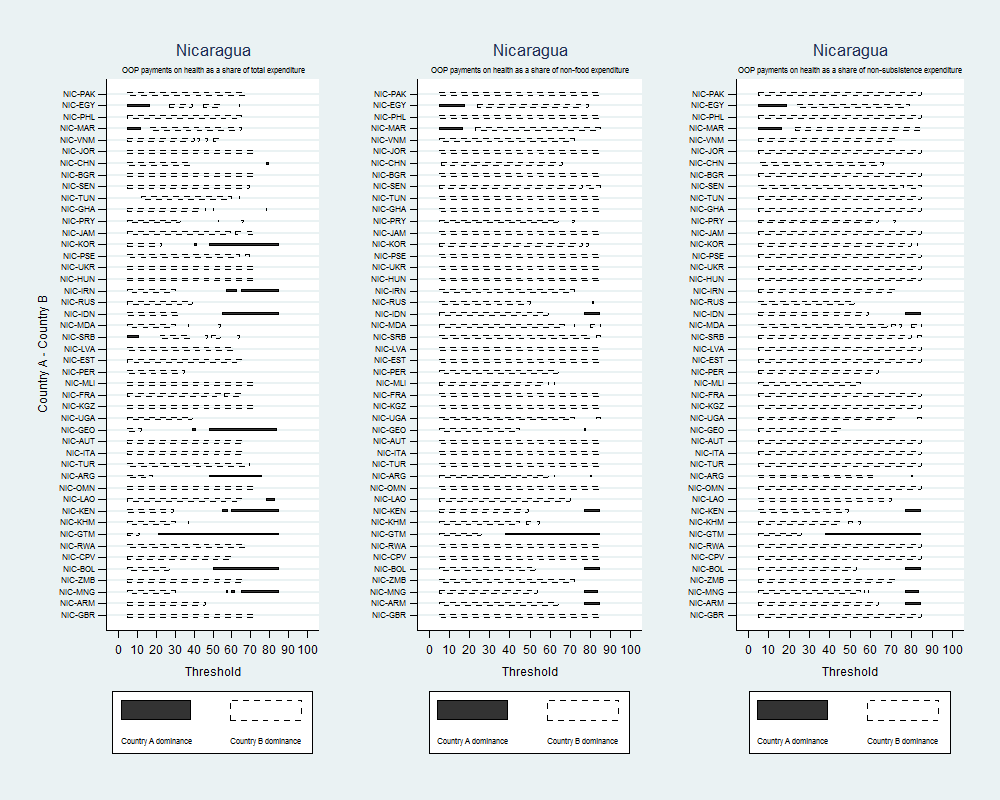


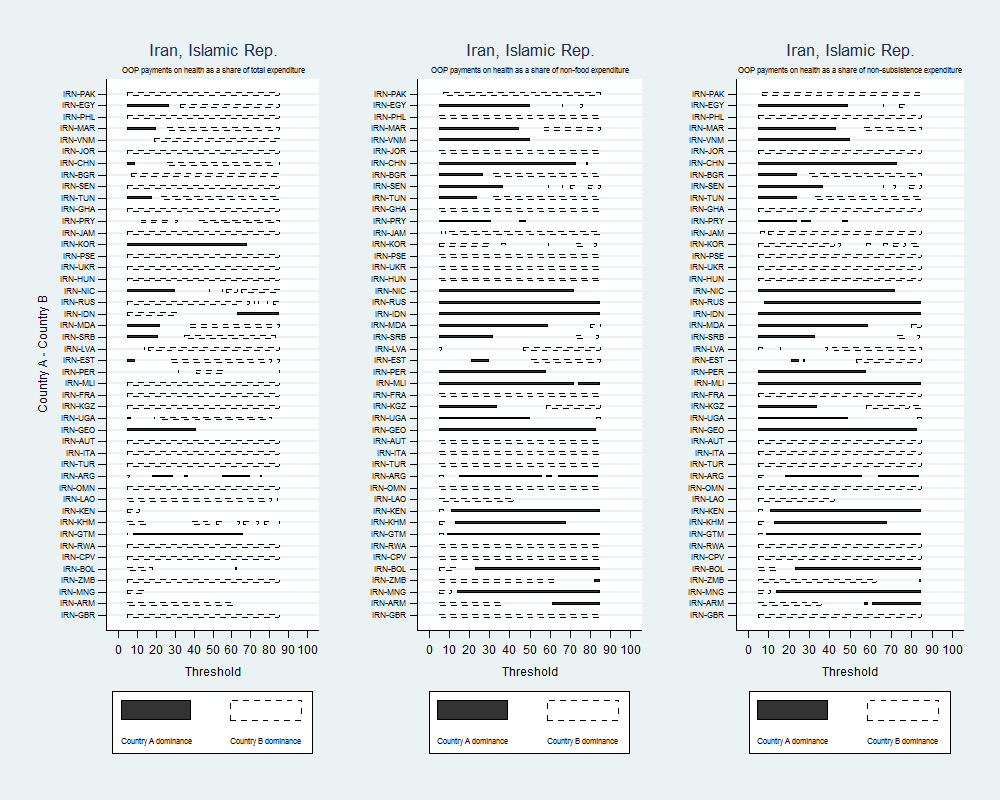


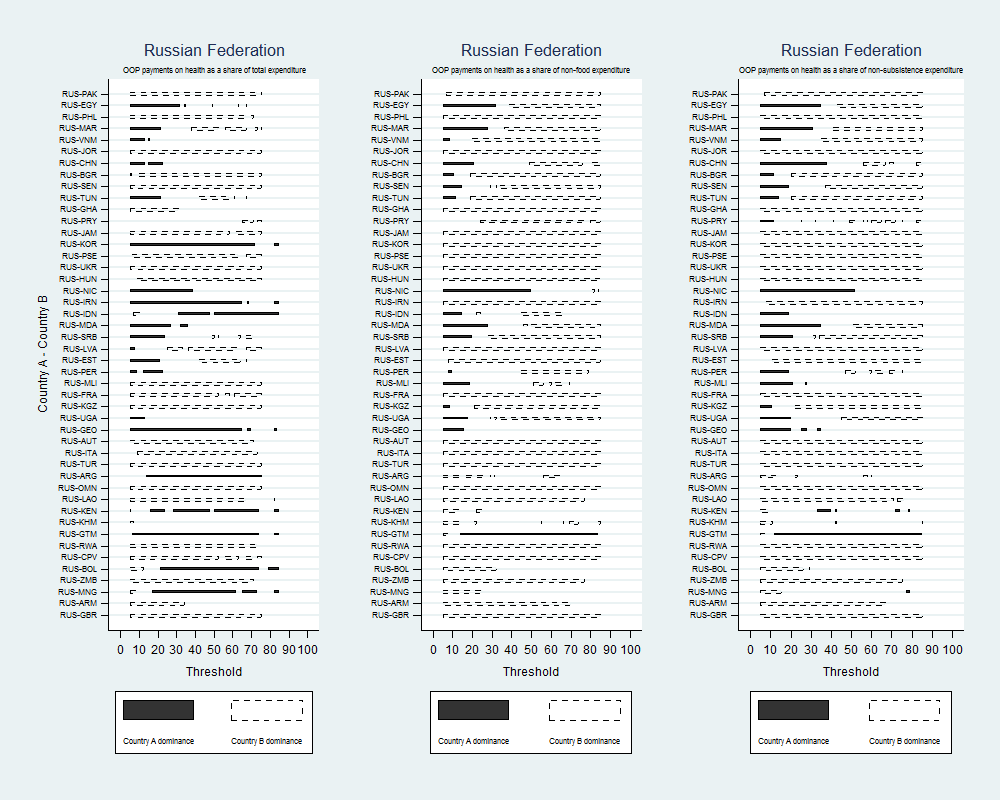


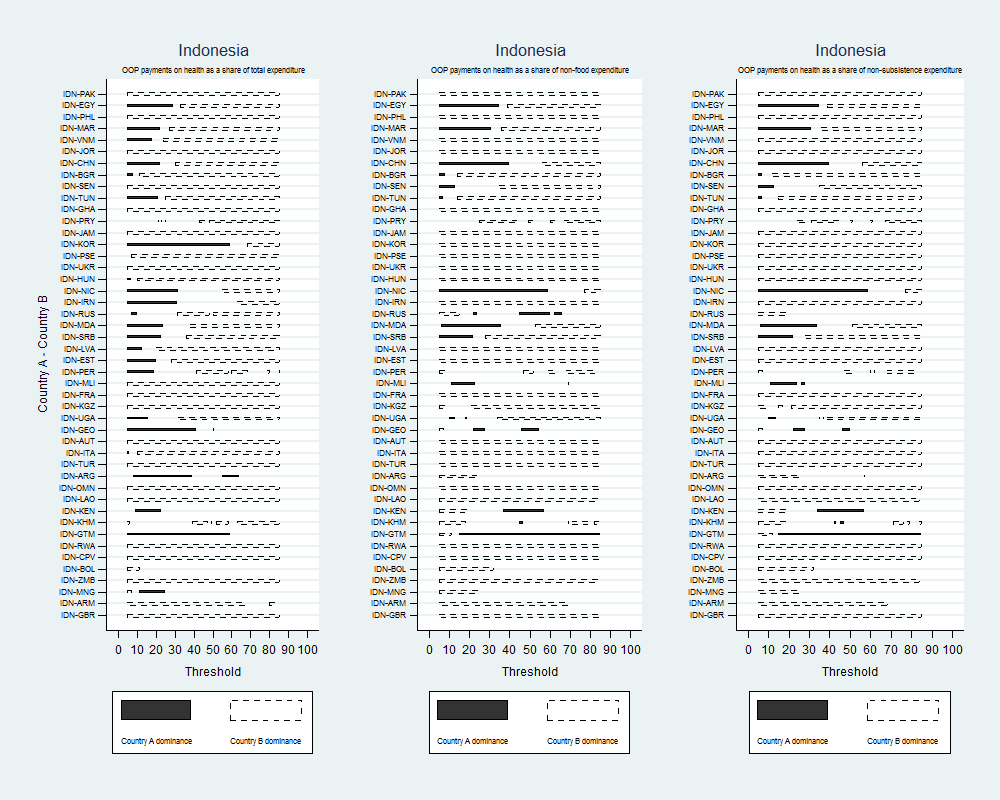


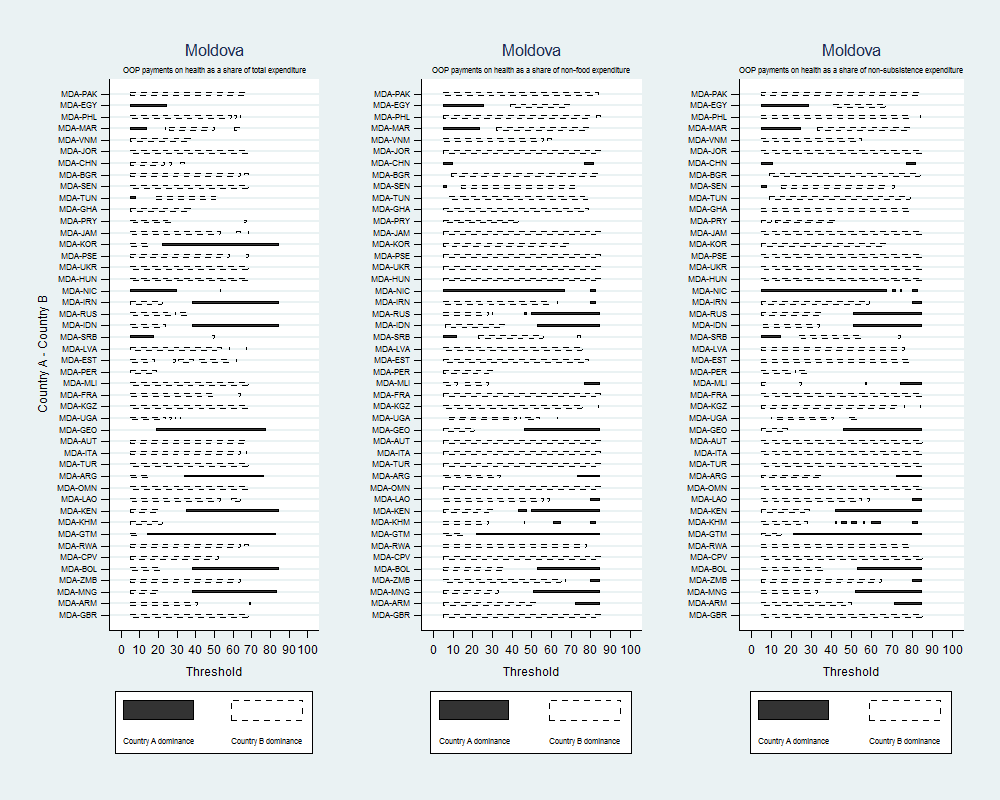


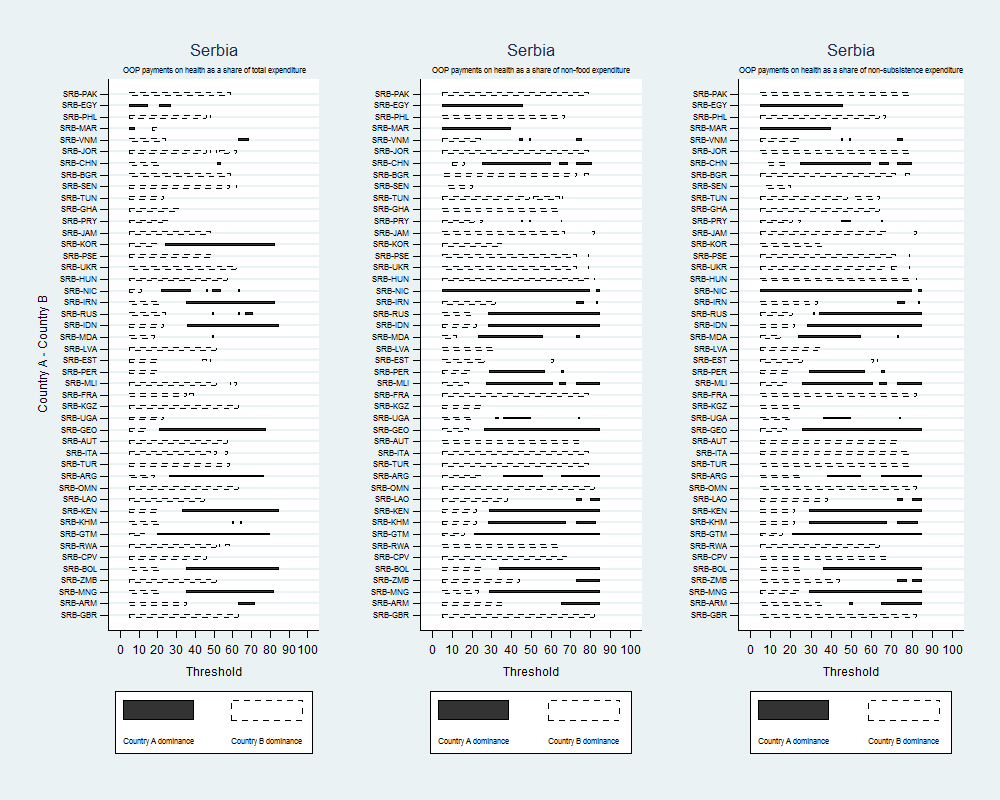


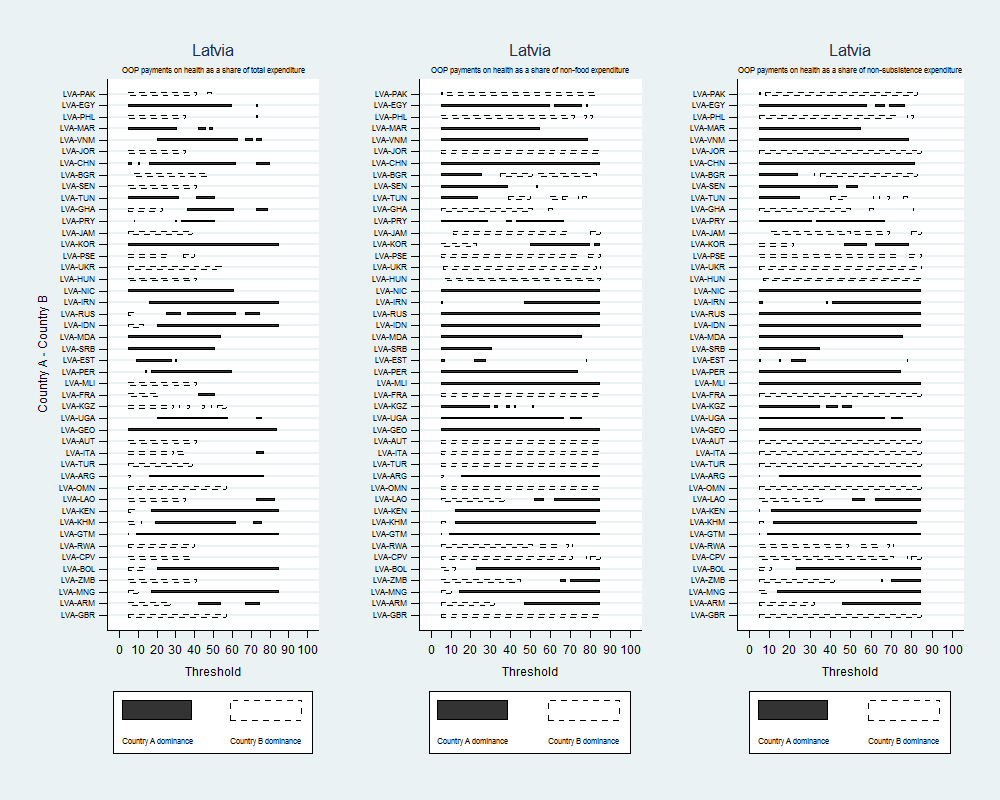


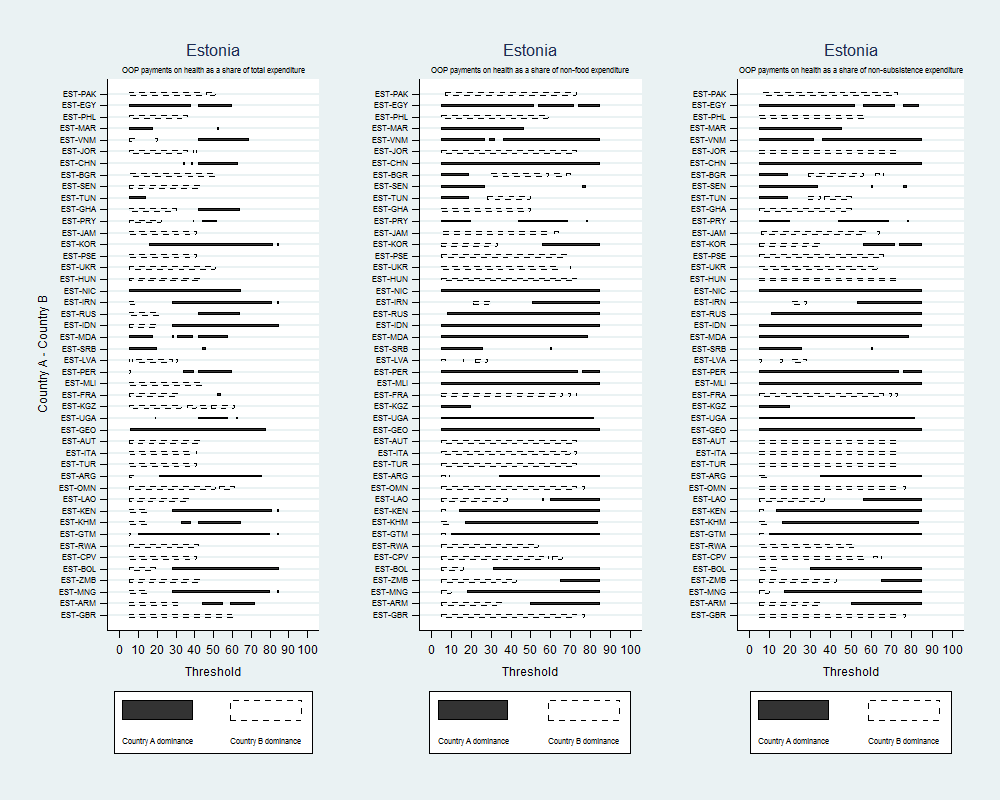


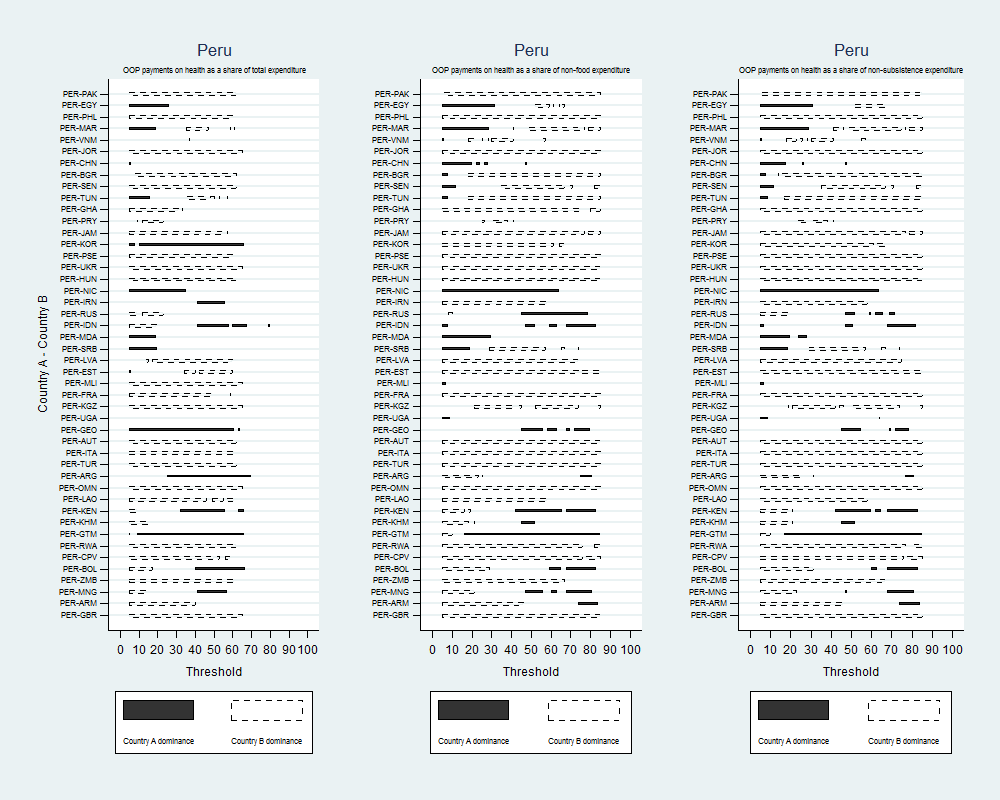


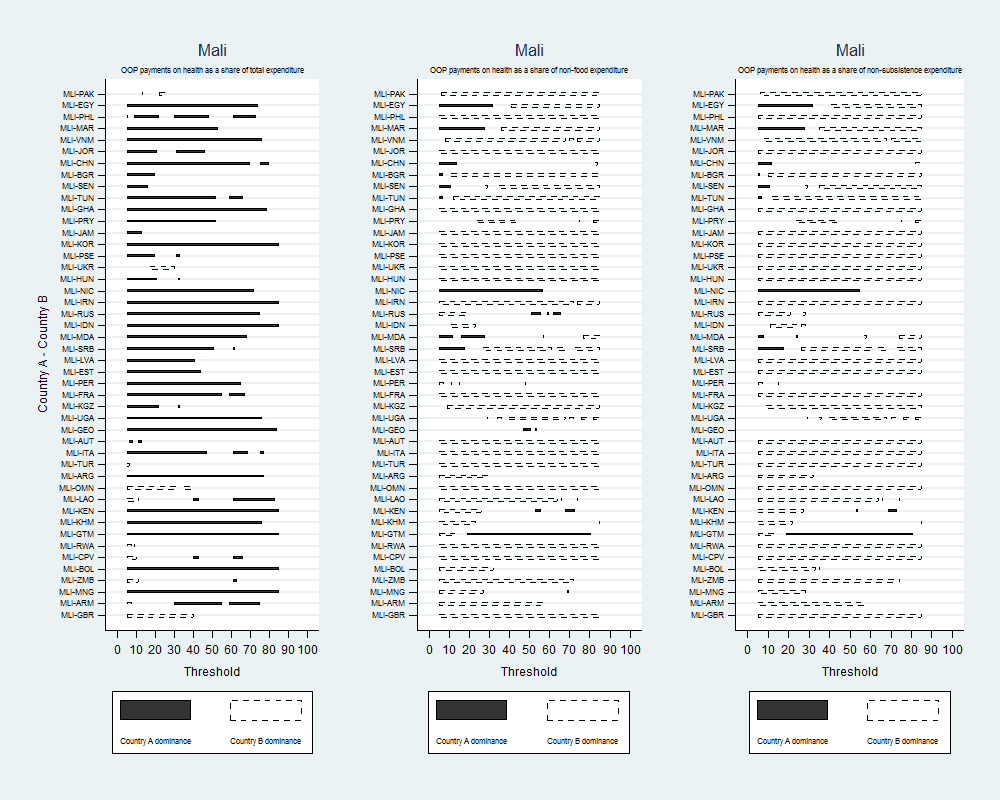


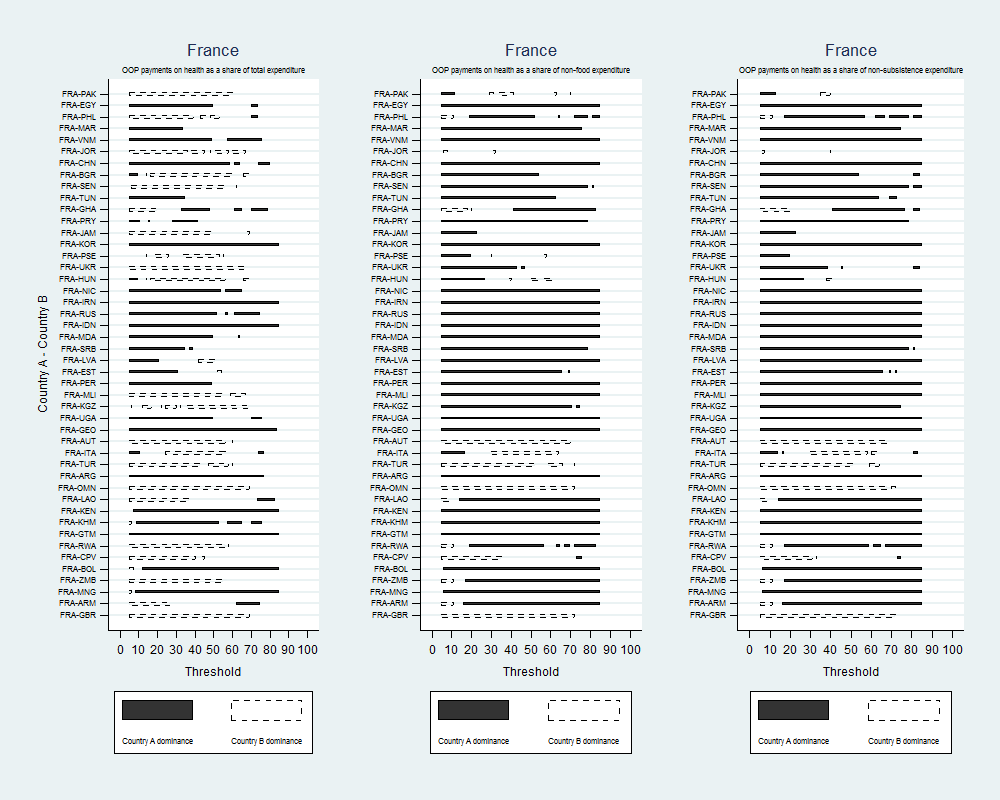


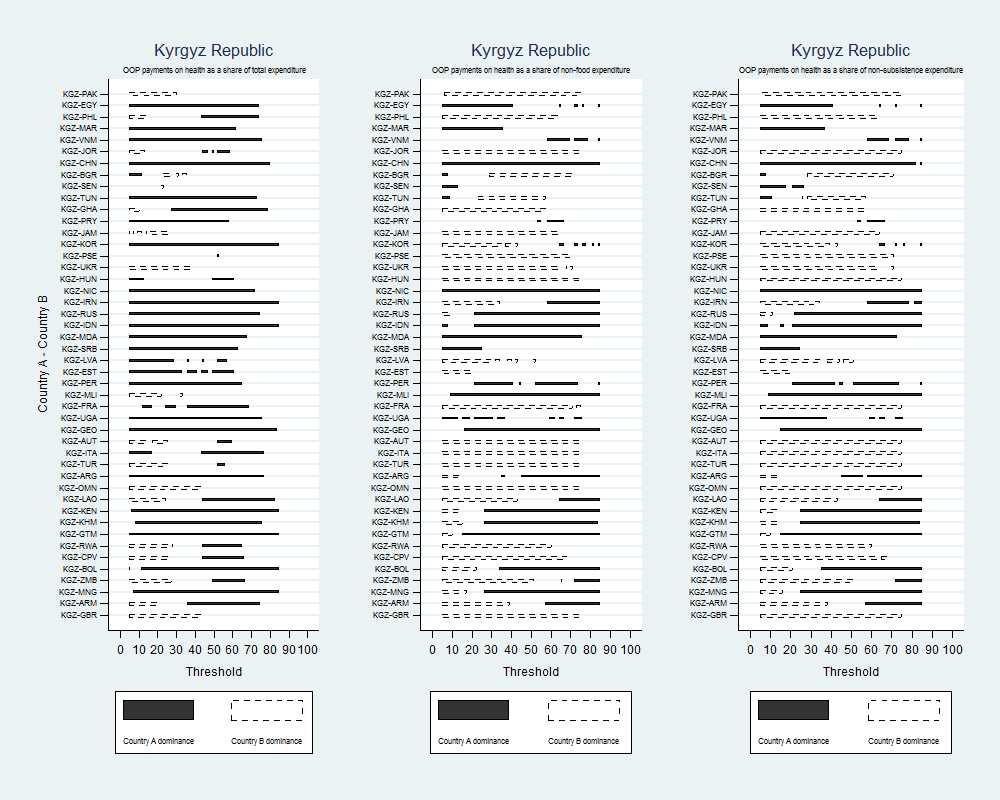


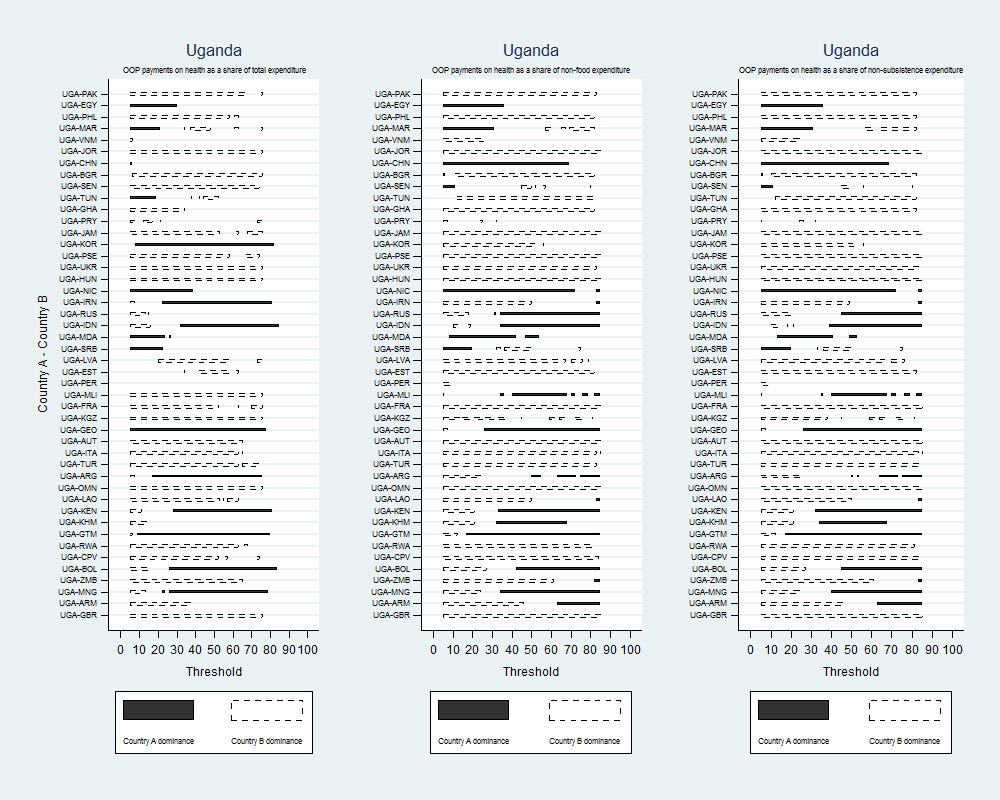


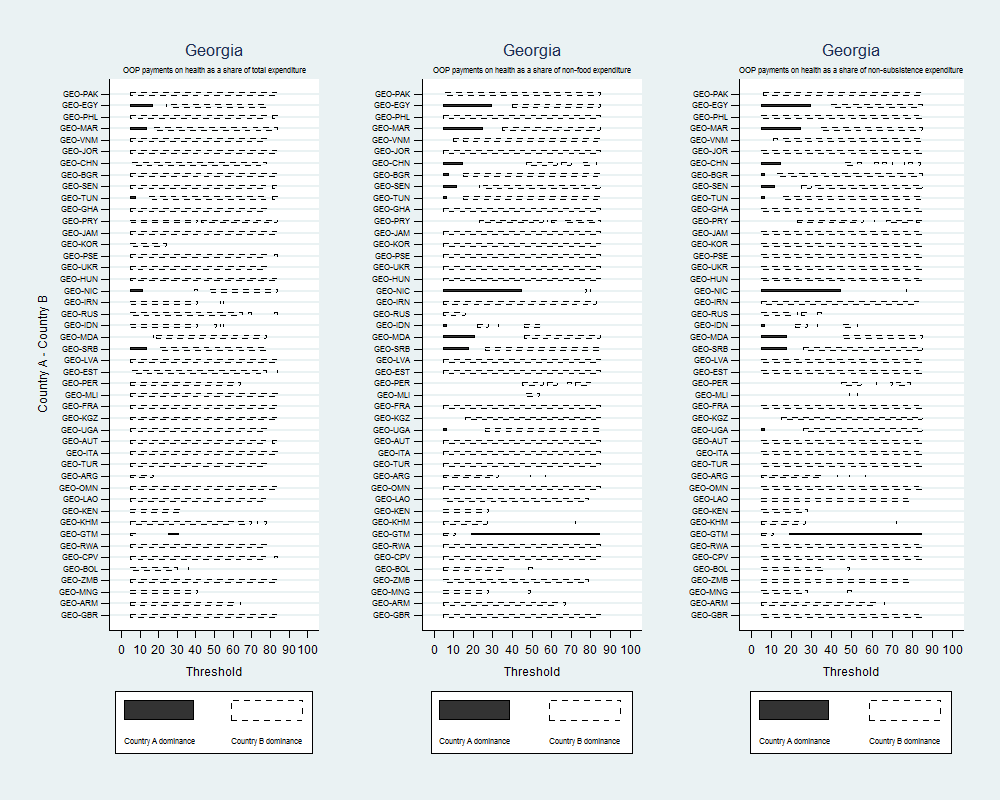


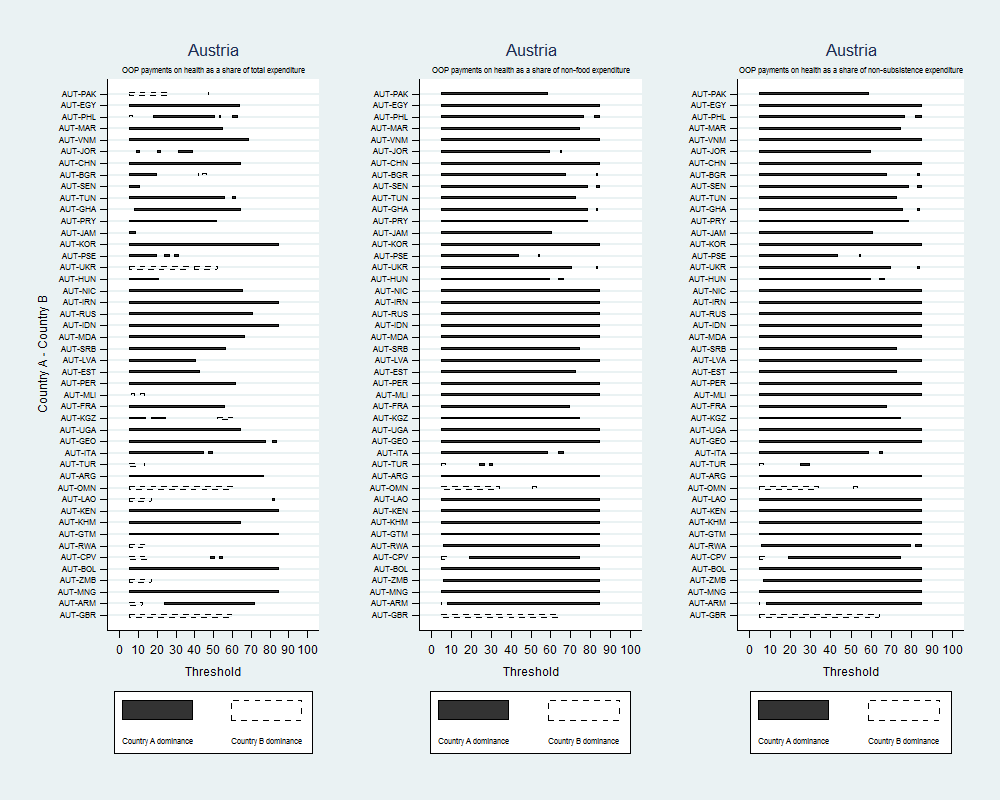


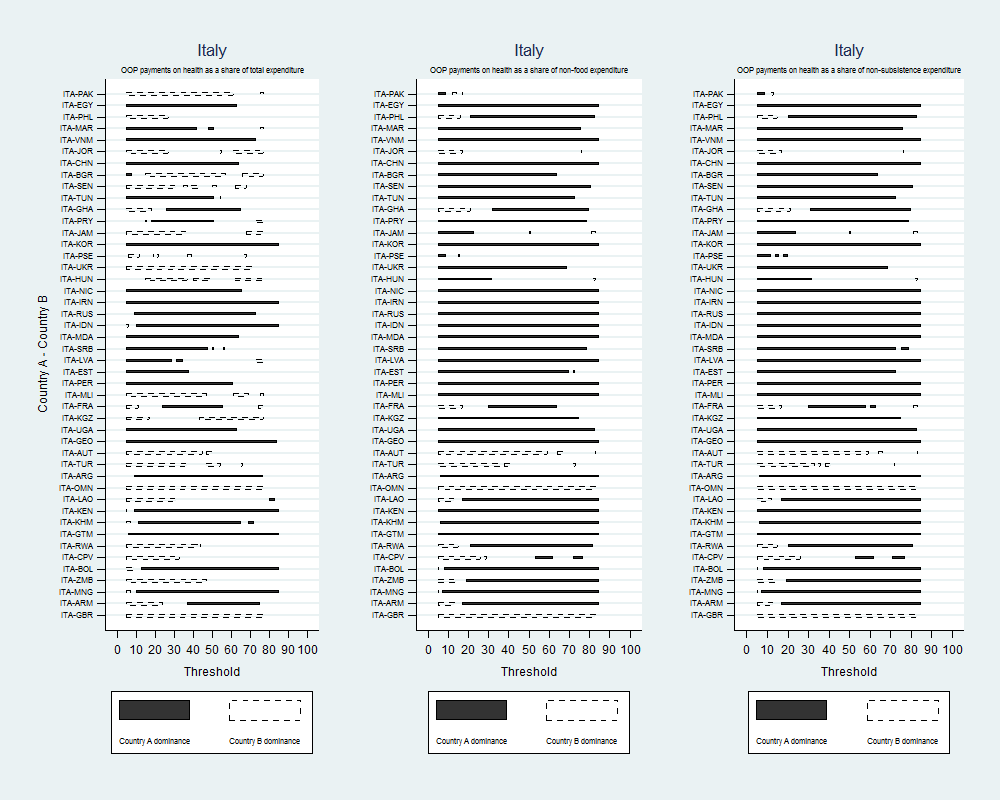


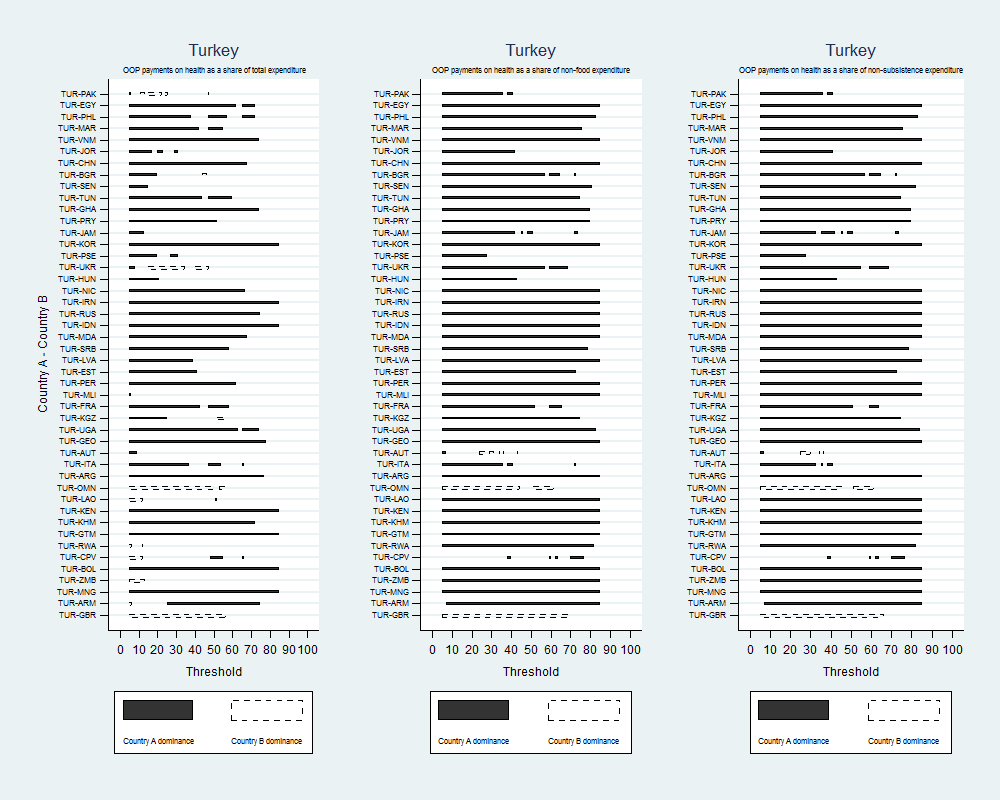


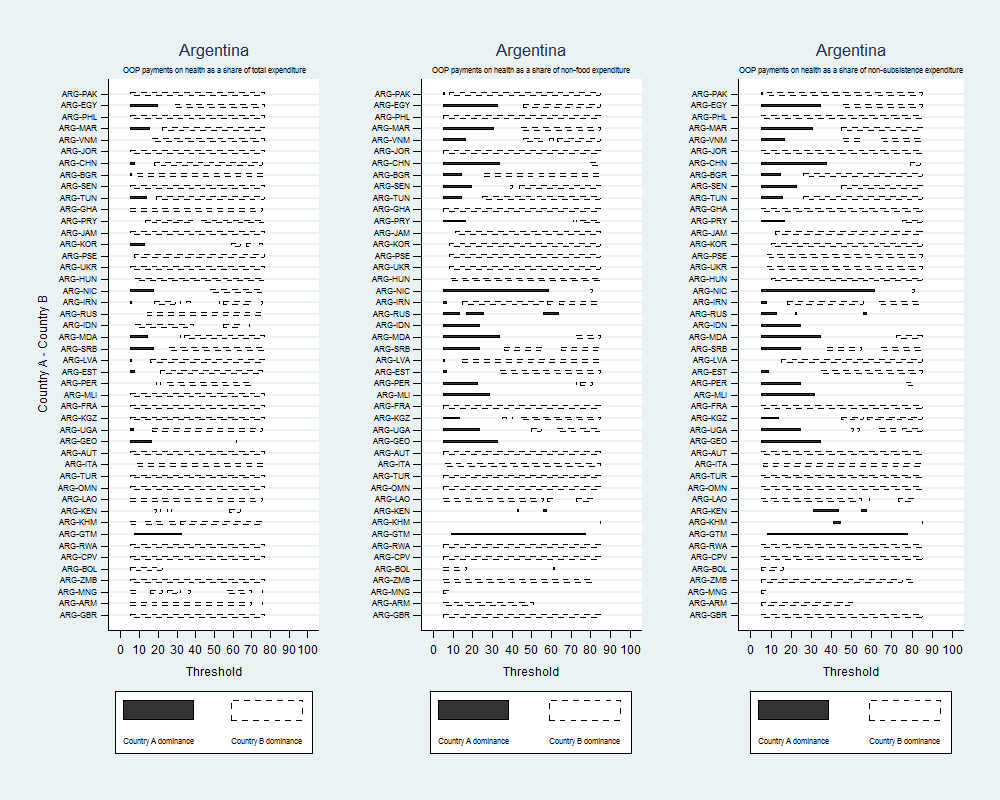


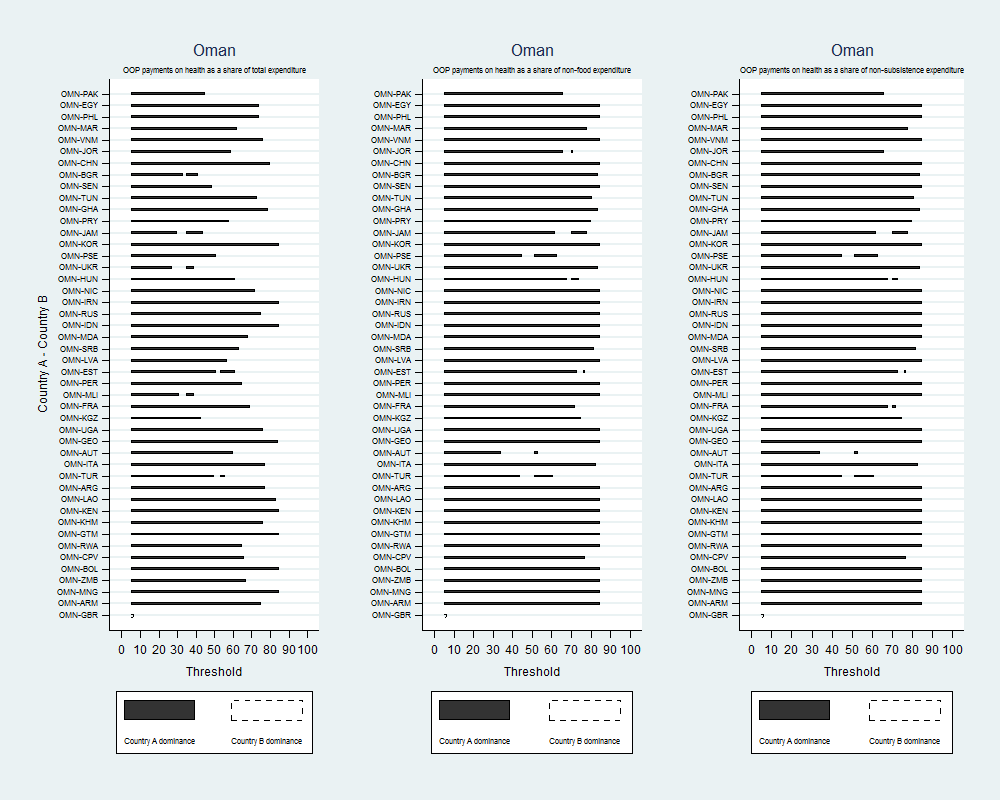


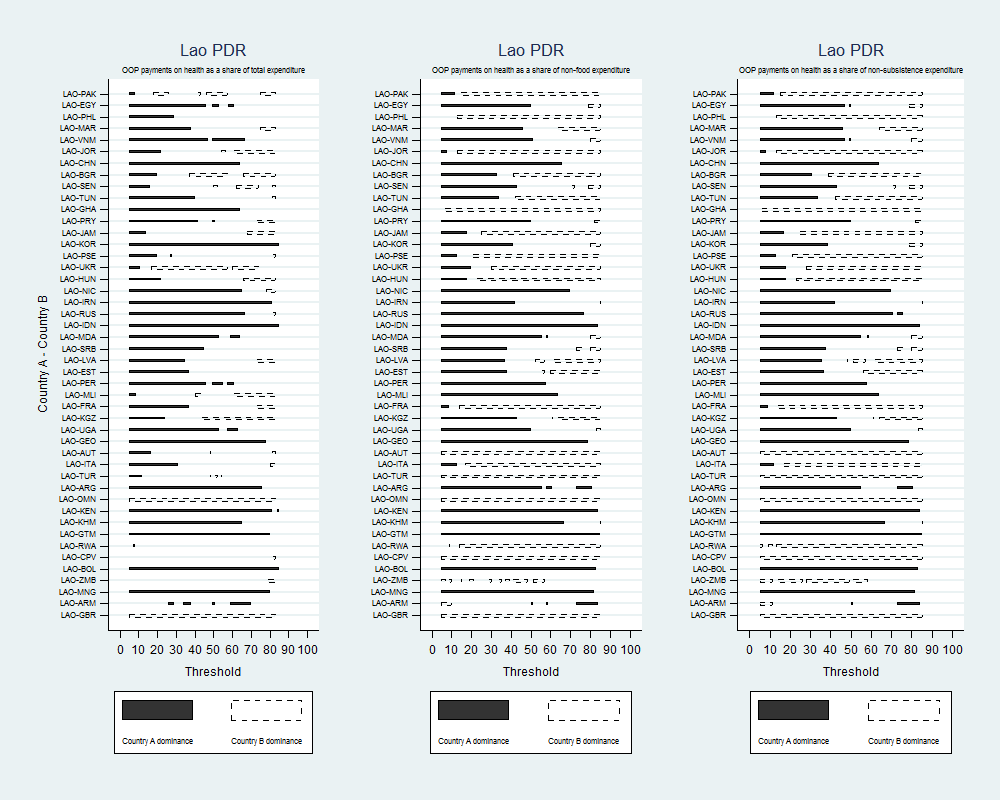


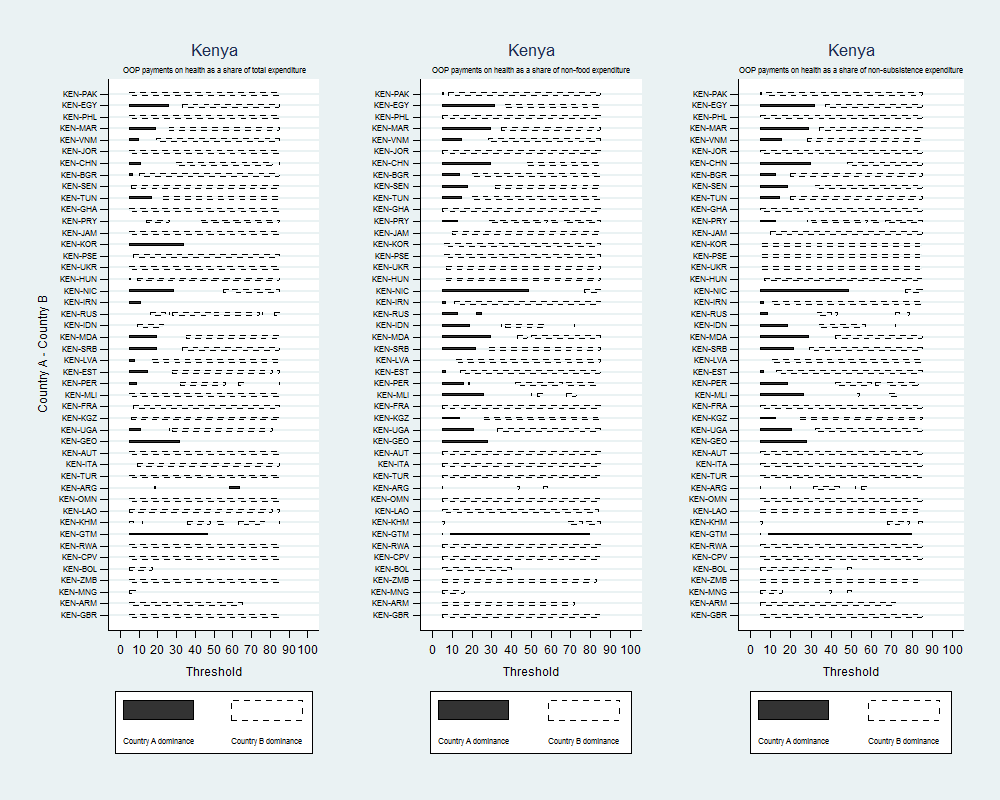


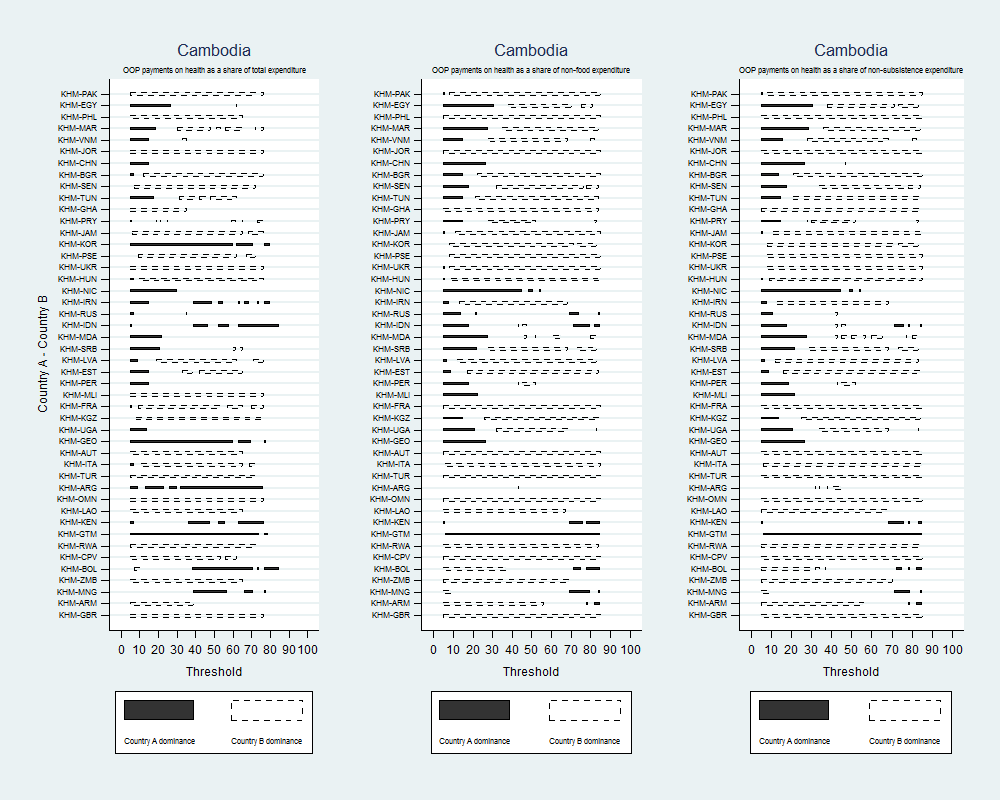


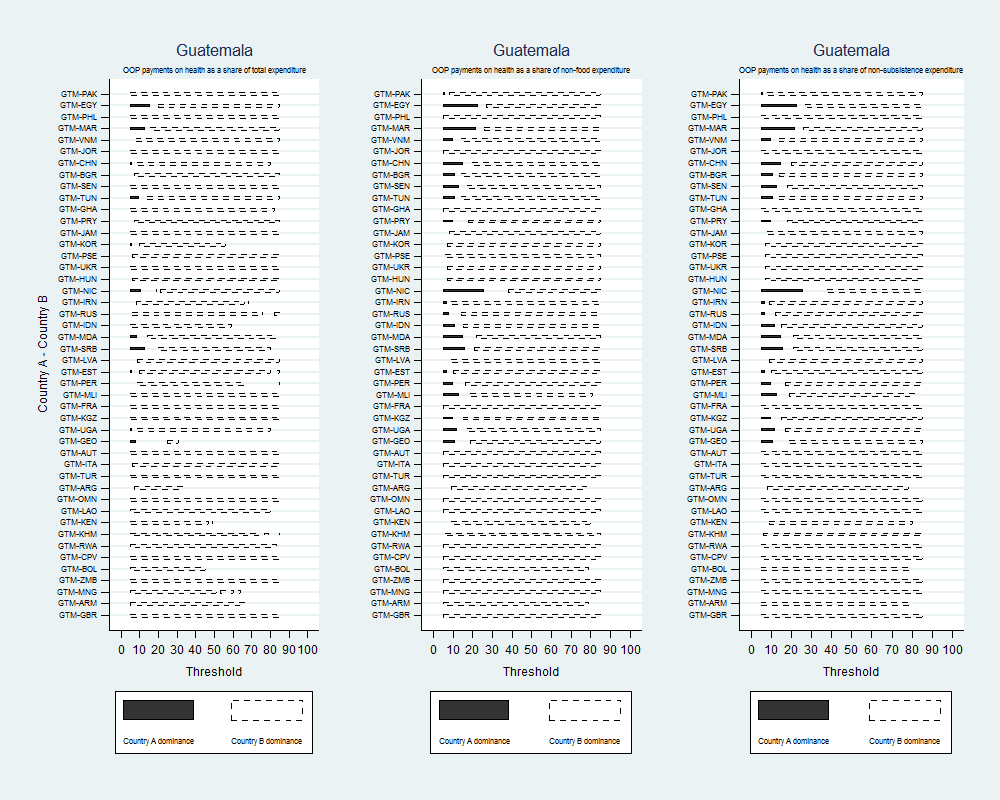


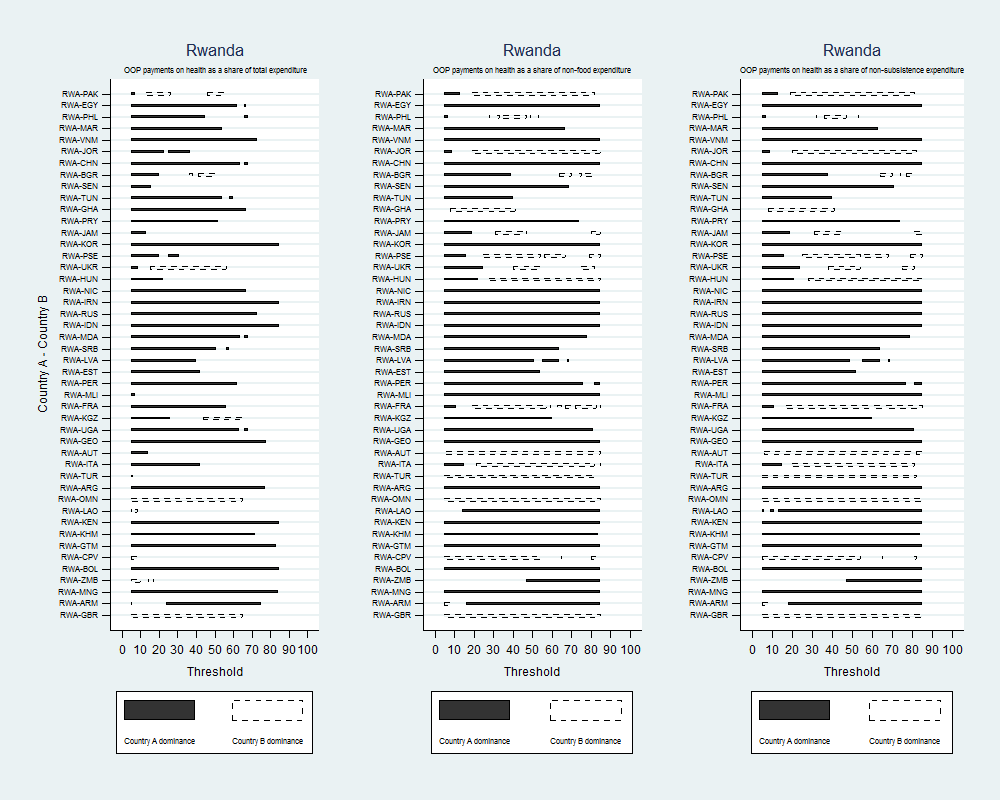


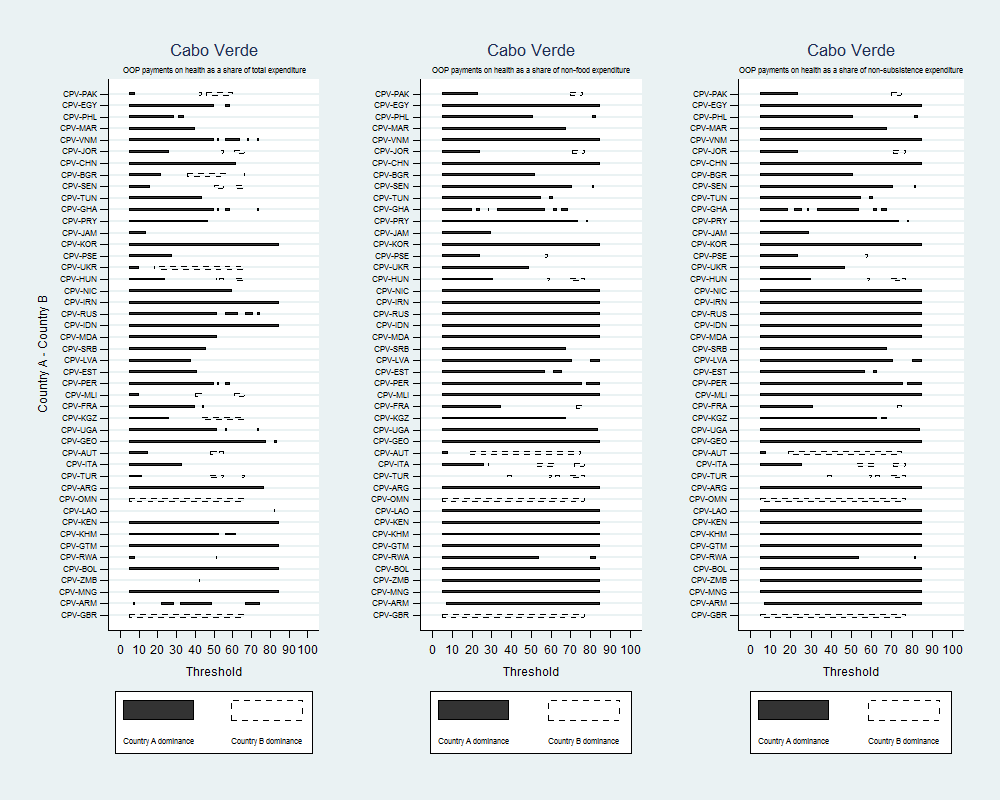


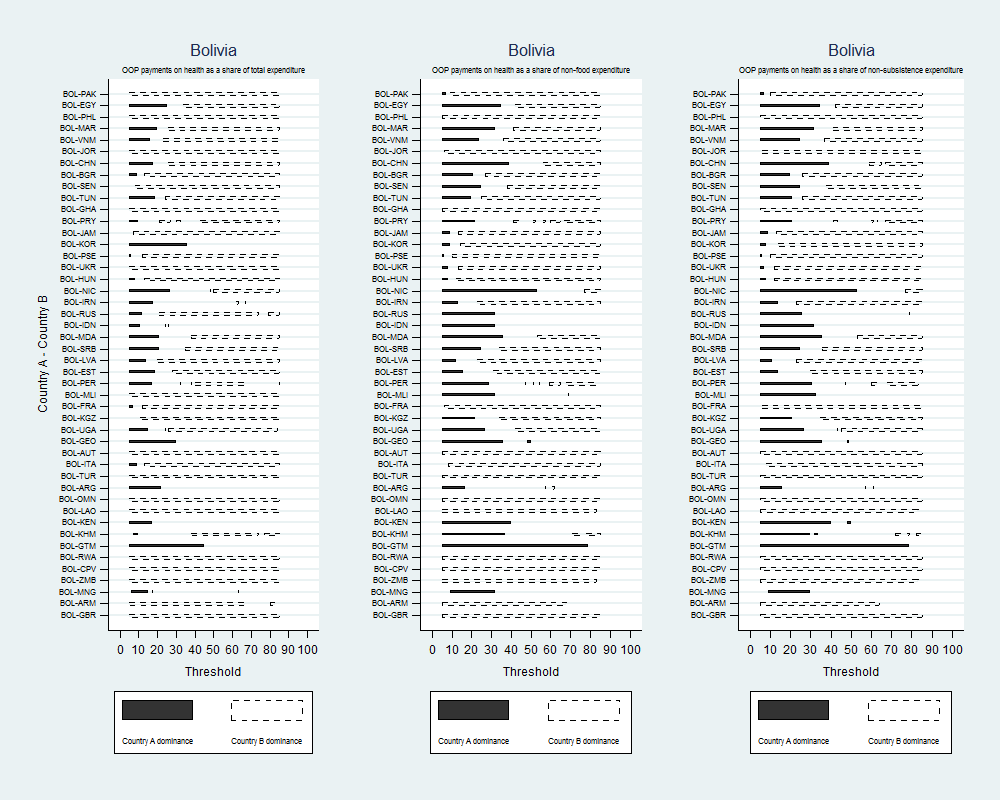


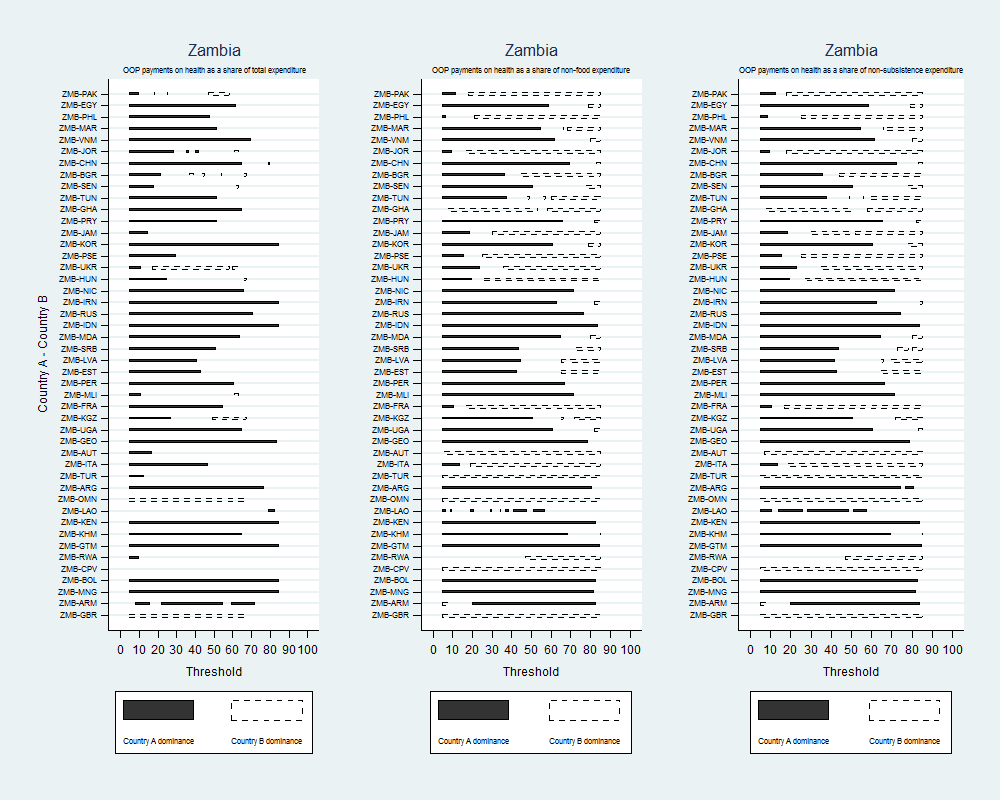


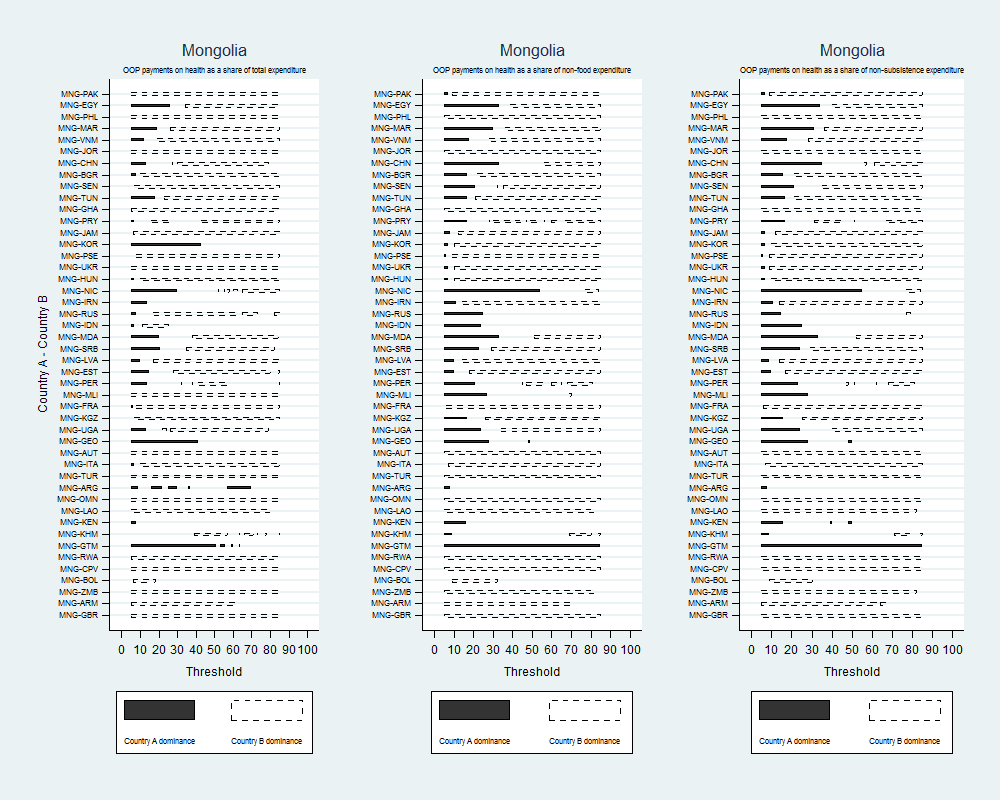


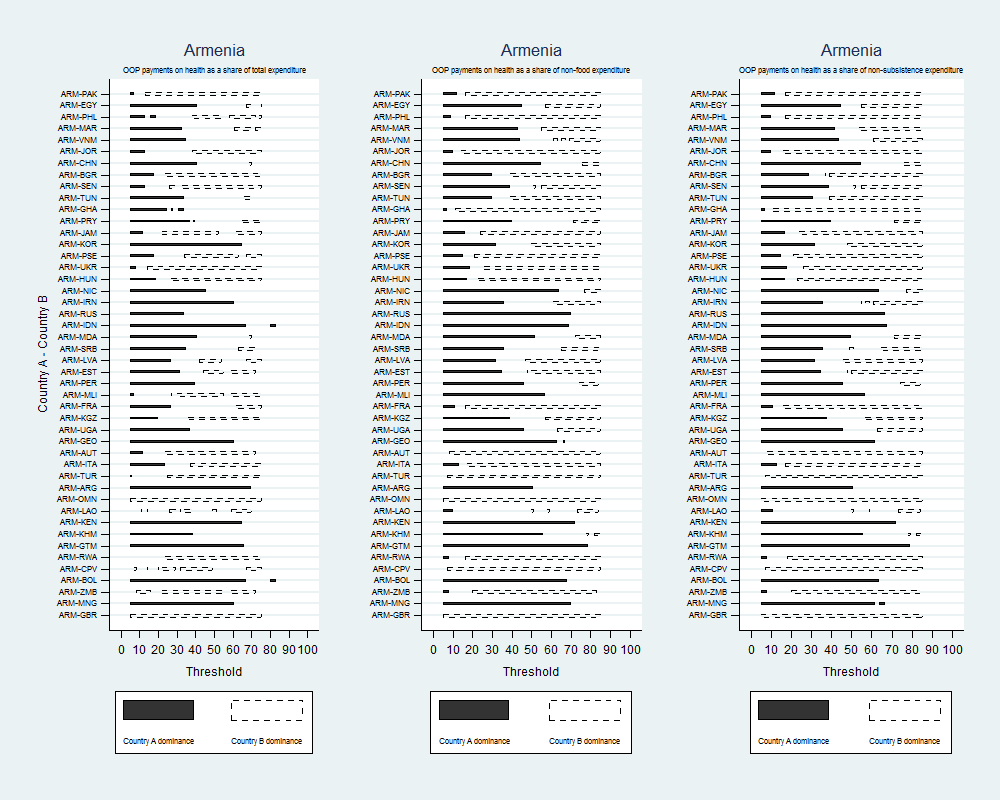


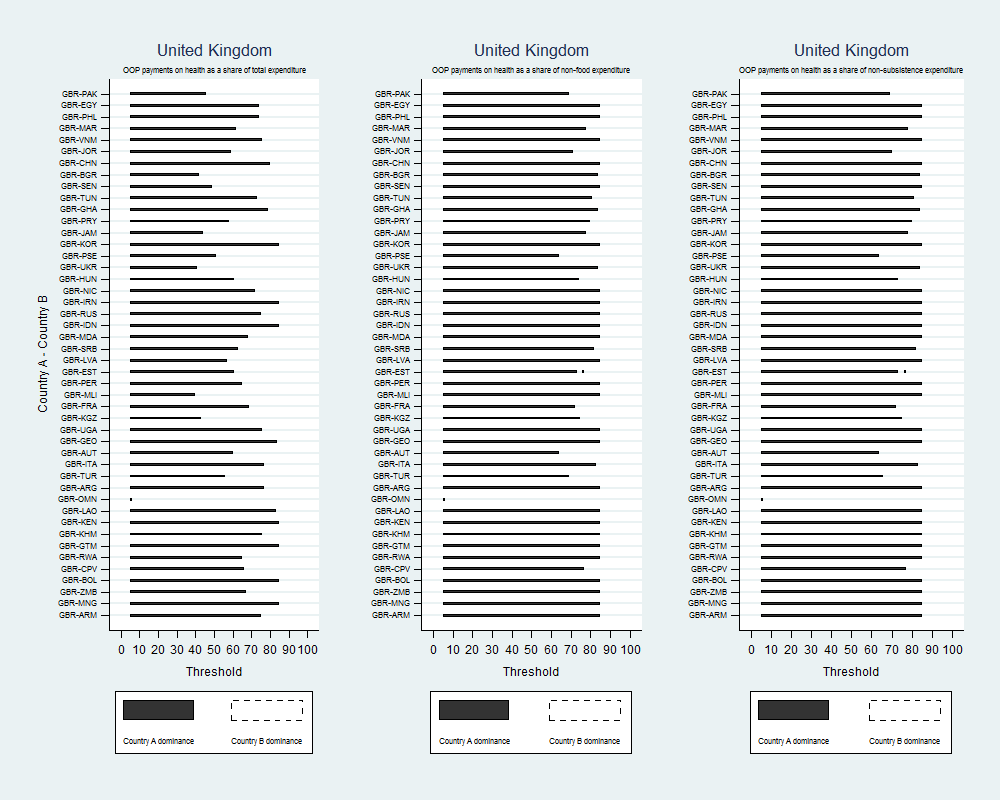

Supplement: Supplementary file 3 — Results of restricted dominance tests. (DOCX 3131 kb) [file 12939_2018_749_MOESM3_ESM.docx]
